# Identifying Potential Factors Associated With Racial Disparities in COVID-19 Outcomes: Retrospective Cohort Study Using Machine Learning on Real-World Data

**DOI:** 10.2196/54421

**Published:** 2024-09-26

**Authors:** Osama Dasa, Chen Bai, Ruba Sajdeya, Stephen E Kimmel, Carl J Pepine, Matthew J Gurka J, Reinhard Laubenbacher, Thomas A Pearson, Mamoun T Mardini

**Affiliations:** 1 Department of Epidemiology College of Public Health and Health Professions and College of Medicine University of Florida Gainesville, FL United States; 2 Division of Cardiovascular Medicine Department of Medicine University of Florida Gainesville, FL United States; 3 Department of Health Outcomes and Biomedical Informatics University of Florida Gainesville, FL United States; 4 Department of Public Health Sciences School of Medicine University of Virginia Charlottesville, VA United States; 5 Laboratory for Systems Medicine Division of Pulmonary, Critical Care, and Sleep Medicine, Department of Medicine University of Florida Gainesville, FL United States

**Keywords:** health disparities, racial disparities, COVID-19 outcomes, social determinants of health, area deprivation index, health outcomes, machine learning, real-world data, COVID-19, SARS-CoV-2, real-world data, socioeconomic status

## Abstract

**Background:**

Racial disparities in COVID-19 incidence and outcomes have been widely reported. Non-Hispanic Black patients endured worse outcomes disproportionately compared with non-Hispanic White patients, but the epidemiological basis for these observations was complex and multifaceted.

**Objective:**

This study aimed to elucidate the potential reasons behind the worse outcomes of COVID-19 experienced by non-Hispanic Black patients compared with non-Hispanic White patients and how these variables interact using an explainable machine learning approach.

**Methods:**

In this retrospective cohort study, we examined 28,943 laboratory-confirmed COVID-19 cases from the OneFlorida Research Consortium’s data trust of health care recipients in Florida through April 28, 2021. We assessed the prevalence of pre-existing comorbid conditions, geo-socioeconomic factors, and health outcomes in the structured electronic health records of COVID-19 cases. The primary outcome was a composite of hospitalization, intensive care unit admission, and mortality at index admission. We developed and validated a machine learning model using Extreme Gradient Boosting to evaluate predictors of worse outcomes of COVID-19 and rank them by importance.

**Results:**

Compared to non-Hispanic White patients, non-Hispanic Blacks patients were younger, more likely to be uninsured, had a higher prevalence of emergency department and inpatient visits, and were in regions with higher area deprivation index rankings and pollutant concentrations. Non-Hispanic Black patients had the highest burden of comorbidities and rates of the primary outcome. Age was a key predictor in all models, ranking highest in non-Hispanic White patients. However, for non-Hispanic Black patients, congestive heart failure was a primary predictor. Other variables, such as food environment measures and air pollution indicators, also ranked high. By consolidating comorbidities into the Elixhauser Comorbidity Index, this became the top predictor, providing a comprehensive risk measure.

**Conclusions:**

The study reveals that individual and geo-socioeconomic factors significantly influence the outcomes of COVID-19. It also highlights varying risk profiles among different racial groups. While these findings suggest potential disparities, further causal inference and statistical testing are needed to fully substantiate these observations. Recognizing these relationships is vital for creating effective, tailored interventions that reduce disparities and enhance health outcomes across all racial and socioeconomic groups.

## Introduction

The COVID-19 pandemic has exposed significant racial disparities, with non-Hispanic Black populations experiencing higher incidence rates and worse outcomes than non-Hispanic White populations [1,2]. Previous research has suggested that these disparities might be attributed to a higher burden of comorbidities in non-Hispanic Black populations [3]. Additional hypothesized contributing factors include systemic socioeconomic disadvantages and the compounding impact of chronic exposure to social and economic stressors, as well as experiences of marginalization [4,5]. Furthermore, some studies have raised the possibility of biological and genetic determinants as contributory risk factors [6,7].

Analyzing racial health disparities solely through isolated data can lead to misleading interpretations, such as attributing higher disease rates to inherent biological factors or perpetuating racial stereotypes, particularly concerning perceived health behaviors [4]. Similarly, dissecting COVID-19 data by geography demands caution to avoid reinforcing negative stereotypes about marginalized communities and “territorial stigmatization” [8]. In the context of the COVID-19 pandemic, a comprehensive framework is necessary that considers social determinants of health (SDOH)—social, economic, and geographical—to properly address the complexities surrounding disparities in outcomes [9-11]. Gathering data at both the neighborhood and individual level is crucial. Such an approach dispels myths rooted in racial biology and highlights the significance of socioeconomic factors, discrimination, and location-based risks [4,5].

It has been recognized that the COVID-19 pandemic is a syndemic involving interactions of multiple factors and conditions [12] where advanced data-driven approaches are needed to capture complex underlying association patterns. Machine learning (ML) offers valuable tools for this complex analysis. Unlike traditional methods, ML can handle vast multidimensional datasets, enabling researchers to discern intricate patterns and relationships that would be impossible to discern using conventional statistical methods. Although many studies have used ML in evaluating the outcomes of COVID-19 [13-18], few have applied these techniques specifically to investigate racial disparities [9,19], which often result from complex interplay among multiple stressors. Most studies focused on a single stressor or a single domain of stressors or were purely ecological, with only county-level COVID-19 data [20].

The purpose of this study is to provide a more comprehensive understanding of the factors contributing to these disparities by analyzing large real-world data from the OneFlorida Research Consortium, using 9-digit zip codes to link individuals to measures of SDOH and other geospatial data related to the place of residence. This will give insight into the importance or relevance of each variable (feature) in predicting worse outcomes of COVID-19. By incorporating comorbidities and SDOH, we seek to explore potential reasons behind the worse outcomes of COVID-19 experienced by non-Hispanic Black patients compared with non-Hispanic White patients and how these variables interact in an ML model.

## Methods

### Design and Population

We conducted a retrospective cohort study of patients diagnosed with COVID-19 infection who were followed prospectively for outcomes. We derived patient-level data from the OneFlorida Clinical Research Network, which includes Floridians enrolled in Medicaid, and robust patient-level electronic health record (EHR) data from public and private health care systems [21]. OneFlorida partners encompass hospitals, clinic settings, and physicians, which provide care for 17 million patients across all of Florida’s 67 counties [21,22]. The data query included adult patients (≥18 years old) with laboratory-confirmed COVID-19 diagnoses between December 1, 2019, and April 28, 2021. Then, using 9-digit zip codes, we linked patients to neighborhood and geospatial variables, as established by previous research [20]. These supplementary variables were derived from several external publicly available datasets, enabling a more comprehensive understanding of the impact of socioeconomic and geospatial factors on outcomes of COVID-19.

### Study Variables (Features)

#### Exposure

We used self-reported race as our exposure variable. It is a surrogate for many interconnected socioeconomic and environmental factors. Race is recognized as a social construct, not a biological determinant [23-25].

#### Outcome

We used a combined measure of severe outcomes of COVID-19. This measure includes intensive care unit admission, intubation, and mortality at the time of the first index encounter. Through this composite outcome, we intended to capture a broader overview of the disease’s severity and short-term impacts. We used encounter, procedures, and death common data model domains in OneFlorida to assess outcomes [26].

### Covariates

#### Individual-Level Variables

At the index encounter, we collected sociodemographic data, including age, self-reported sex, race and ethnicity as non-Hispanic Black or non-Hispanic White, and previous insurance status. We also included 9-digit zip codes, BMI, smoking, alcohol, and substance use status.

In line with the Centers for Disease Control and Prevention’s compilation of medical conditions associated with an increased risk for severe outcomes of COVID-19 [27], we examined comorbidities during the encounters from January 1, 2012, leading up to the COVID-19 index encounter. Conditions with a less than 1% prevalence in our study population were excluded. We derived COVID-19–related comorbid conditions by mapping available *International Classification of Diseases, Ninth Revision* and *International Statistical Classification of Diseases, Tenth Revision* codes to the Healthcare Cost and Utilization Project (HCUP) Clinical Classification Software definitions [28]. We also used the well-established Elixhauser Combined Comorbidity Index (ECI) to quantify the aggregate burden of comorbid conditions. This index includes 31 comorbidities and reliably predicts outcomes like in-hospital mortality, length of stay, adverse events, and hospital discharges [29-32].

#### Group-Level Variables

##### Area Deprivation Index

The area deprivation index (ADI) evaluates community deprivation, impacts health outcomes, and guides policy and health care use patterns [33]. It has demonstrated that residing in a disadvantaged area can be as detrimental to health as certain chronic diseases [33]. The Centers for Medicare and Medicaid Services (CMS) leverage the ADI in their strategies [34]. During the COVID-19 pandemic, the ADI informed equitable resource distribution, emphasizing the role of socioeconomic factors in disease outcomes [35,36].

Using the 2018 ADI, we evaluated community-level disparities. Calculated at the United States census block group level, it reflects a “neighborhood” of approximately 600 to 3000 residents [33]. This granularity offers a localized view of health-related social and geospatial determinants. We geocoded patient addresses, assigning an ADI rank based on their Florida residential census block group, sourced from their 9-digit zip codes. Higher ADI ranks signify greater social disadvantage.

##### Geospatial Variables

We used an extensive exposome-wide association study to identify additional external exposome elements that may correlate with COVID-19 mortality. This study included 337 external exposome factors encompassing 9 distinct categories [20]. It identified 4 external exposome factors at the county level significantly associated with worse outcomes of COVID-19. These included variables characterizing the natural (criteria air pollutants and air toxicants), built (food environment), and social environment (vacant land) [20]. The results of this exposome study reaffirm the importance of environmental and geospatial variables in understanding and predicting outcomes of COVID-19. Our analysis incorporated data relating to these 4 variables by linking them to patients’ places of residence in Florida (Table 1).

**Table 1:** Data sources, time periods, and spatial scales of environmental measures.

Spatial variable	Data source	Time period	Spatial scale	Temporal scale
Particulate matter 2.5	Atmospheric composition analysis group, WUSTL^a^ [37]	2006-2018	0.01 degree in lon and lat	1 year
Nitrogen dioxide (air pollution)	The center for air, climate, and energy solutions [38]	2006-2015	Census block group	1 year
Percent students eligible for reduced-price lunch, 2015 (food environment)	Food environment atlas [39]	2007-2018	County	Cross-sectional
Percent addresses in the previous quarter with no-stat currently in service (vacant land)^b^	Aggregated USPS^c^ administrative data on address vacancies, HUD^d^ [40]	2006-2019	Census tract	3 months

^a^WUSTL: Washington University in St Louis.

^b^Total no-stat addresses are the addresses that can be classified as “No-Stat” for many reasons, including, (1) rural route addresses that are vacant for 90 days or longer and (2) addresses for businesses or homes under construction and not yet occupied addresses.

^c^USPS: United States Postal Service.

^d^HUD: US Department of Housing and Urban Development.

This table outlines the data sources, time periods, and spatial scales of various environmental measures used in our retrospective cohort study of COVID-19 patients from the OneFlorida Clinical Research Network. These environmental variables, sourced from diverse national databases, were analyzed at different spatial and temporal scales to assess their impact on COVID-19 outcomes among Floridians. The study encompasses adult patients (≥18 years old) with confirmed COVID-19 diagnoses between December 1, 2019, and April 28, 2021.

##### Statistical Analysis

Categorical variables were reported as frequencies and percentages, while continuous variables were reported as means with SDs or medians with IQR. We used the Chi-square test for categorical variables. For continuous variables, we used the *t* test for normally distributed data or the Wilcoxon-Mann-Whitney *U* test when data were not normally distributed. Appropriate transformations were applied to certain continuous variables with skewness to achieve a normal distribution. Following this, *z* score standardization was used to establish a standard scale for interfactor comparison. We also evaluated mixed correlation coefficients between all variables included in our models.

K-nearest neighbors imputation was used when missing data was present. This technique estimates the missing values based on attributes of the most similar observations, where similarity is calculated using a distance function [41]. K-nearest neighbors imputation is frequently regarded as a more robust and sensitive method for missing value estimation than conventional techniques [42].

##### Machine Learning Model Development

We used Extreme Gradient Boosting (XGBoost), a robust ML framework known for its efficiency, flexibility, and portability [43]. It is an ensemble learning algorithm based on the gradient boosting framework, in which models are built sequentially to boost (increase) the performance of the previous models by using the gradient descent algorithm to minimize errors [43]. Our selection of XGBoost is based on simplified interpretability and the inclusion of feature selection as part of the model-building process. XGBoost exhibits various advantages that render it a compelling alternative to conventional statistical techniques and other ML algorithms.

**Figure 1:**
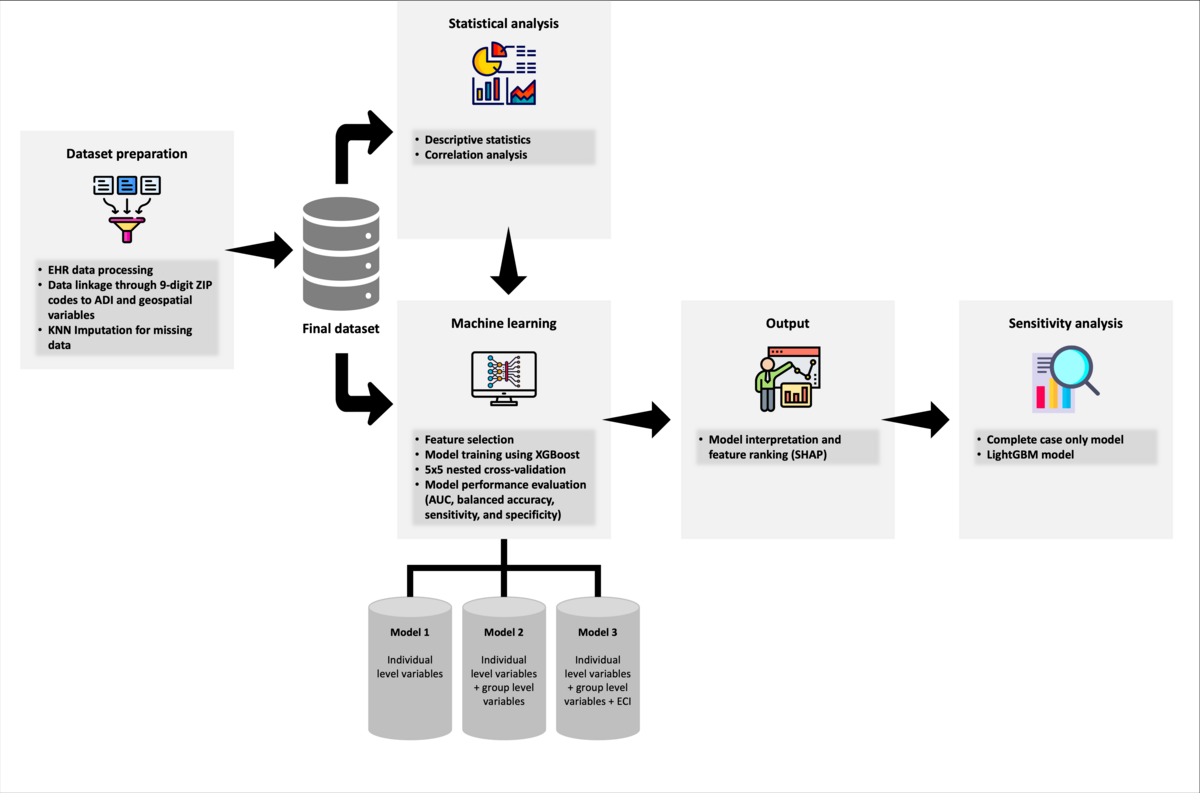
Study flowchart and machine learning analytical framework. ADI: area deprivation index; AUC: area under the receiver operating characteristic curve; EHR: electronic health record; KNN: k-nearest neighbors; SHAP: Shapley Additive Explanations.

We developed and validated 3 consecutive ML models to improve the understanding of the models’ operational dynamics and the incremental contribution of different sets of variables to the outcome. Model 1 consisted exclusively of individual-level variables, which included sociodemographic data and individual comorbidities. Following this, the model was extended to encompass variables at the group level; specifically, we incorporated ADI and environmental measures (particulate matter 2.5 [PM_2.5_], nitric oxide [NO_2_], vacant land measure, and food environment measure), resulting in the development of model 2. In our final model, model 3, instead of individual comorbidities, we incorporated the ECI while preserving the group-level variables from model 2. Implementing a unidimensional singular numerical summary of comorbidities facilitates the modeling and integration with other covariates instead of requiring the modeling and interactions between covariates and each constituent of the comorbidity score, thereby enhancing computational efficiency. All models incorporated baseline variables (age, sex, and BMI; Figure 1).

The figure illustrates the study flowchart and the machine learning analytical framework used in the retrospective cohort study of COVID-19 patients from the OneFlorida Clinical Research Network. It provides a visual summary of the study design, data sources, patient selection criteria, and the ML models used to analyze the data, capturing adult patients (≥18 years old) diagnosed with COVID-19 between December 1, 2019, and April 28, 2021.

In addition, to ensure a comprehensive understanding of our models’ performance, we initiated our analysis by constructing a global model that includes the entire population. Subsequently, we created individualized models for non-Hispanic White and non-Hispanic Black populations across all 3 models. This allowed us to compare the relative importance of features within and between each demographic group.

##### Model Performance and Evaluation

We used nested cross-validation (CV) to evaluate our ML models with 5 outer and inner folds (55 nested CV). A nested CV can help ensure rigor and enhance confidence in model generalizability and scalability [44] (Methods S1 in Multimedia Appendix 1 [44-47]). We calculated and reported the following measures of the models’ performance: the average value and SD of the area under the receiver operating characteristic curve (AUC), balanced accuracy, sensitivity, and specificity from the 5 outer folds (Figure 1). AUC measures the overall discriminative ability, balanced accuracy gauges fair assessment across classes, and sensitivity and specificity offer insight into the model’s prediction accuracy for each class.

##### Model Interpretation and Feature Ranking

Interpreting complex models like XGBoost can be challenging due to their reliance on numerous decision trees, making it difficult to intuit the relationships between features and outcomes. To address this, we used Shapley Additive Explanations (SHAP) values, a method rooted in coalitional game theory [48], which decomposes individual predictions to quantify the influence of each feature (Methods S2 in Multimedia Appendix 1 [44-47]) [45].

Finally, to assess the robustness of our findings, we conducted a sensitivity analysis using a complete-cases-only approach, excluding cases with missing data and without resorting to data imputation (model 4). We also assessed the potential for divergent feature importance rankings with alternative models using light gradient boosting machine (LightGBM) algorithm [49]. It is another high-performance, gradient-boosting framework that uses tree-based learning algorithms (Figure 1) [49]. Data analyses were performed using (R version 3.6.1; R Core Team) and Python (Version 3.7; Python Software Foundation). The data preprocessing, imputation, and grid search steps were implemented using the Python Sklearn package. The XGBoost algorithm was implemented using the XGBoost package. We followed Enhancing the Quality and Transparency of Health Research Network guidelines for reporting ML analyses in observational studies [50,51].

### Ethical Considerations

This study used a deidentified electronic health record dataset provided by the OneFlorida Clinical Research Consortium. The data were deidentified before analysis to ensure participant privacy and confidentiality, with no identifying information accessible to the researchers. The study was reviewed and approved by the Institutional Review Board at the University of Florida (Institutional Review Board 202001531), which determined that informed consent was not required as this research involved secondary analysis of existing, deidentified data. No compensation was provided to participants as the study did not involve direct interaction with individuals. In addition, no images or supplementary materials in this manuscript could potentially identify individual participants.

## Results

Our initial data collection included 49,461 patients, of which 35% (n=17,311) were non-Hispanic White and 22.8% (n=11,277) were non-Hispanic Black. After limiting our study to only non-Hispanic White and non-Hispanic Black populations, the final sample size for this analysis was 28,943 patients. Compared with non-Hispanic White patients, non-Hispanic Black patients tended to be younger; had higher BMI; had fewer outpatient visits; and had higher rates of no insurance, visits to the emergency department, and hospital admissions (Table 2). In the non-Hispanic Black group, the burden of comorbidities and the total ECI scores were significantly higher, especially hypertension, coronary artery disease, congestive heart failure (CHF), and chronic kidney disease. Geospatial and neighborhood variables revealed that non-Hispanic Black populations resided in regions with higher (hence less favorable) ADI rankings, NO_2_, and PM_2.5_ concentrations, as well as lower vacant land and food environment measures. Finally, the non-Hispanic Black group experienced the highest rates of the composite primary outcome (Table 2). Correlation analysis revealed moderately high correlations when insurance status was compared with PM_2.5_ and NO_2_ concentrations. Unsurprisingly, there were also high correlations between ECI scores and most comorbidities (Figure S1 in Multimedia Appendix 1 [44-47]).

**Table 2:** Baseline characteristics and outcomes of the study population stratified by race^a^. The table presents the demographic characteristics of adult patients (≥18 years old) diagnosed with COVID-19 within the OneFlorida Clinical Research Network between December 1, 2019, and April 28, 2021.

		Non-Hispanic White (n=17,651)	Non-Hispanic Black (n=11,293)	Total (N=28,944)
Age, (years), mean (SD)		52.82 (20.24)	48.16 (18.22)	51 (19.61)
Sex (female), n (%)		9490 (53.8)	6791 (60.1)	16,281 (56.3)
BMI, kg/m^2^, mean (SD)		29.96 (7.66)	33.19 (9.22)	31.31 (8.50)
**Insurance status, n (%)**				
	Medicare or private	15,494 (87.8)	8591 (76.1)	24,085 (83.2)
	Medicaid or no insurance	1566 (8.9)	2191 (19.4)	3757 (13.0)
	Unknown	589 (3.3)	511 (4.5)	1100 (3.8)
**Encounter type, n (%)**				
	Outpatient	7476 (42.4)	2712 (24)	10,188 (35.2)
	ED^b^	4551 (25.8)	4525 (40.1)	9076 (31.4)
	Inpatient	4884 (27.7)	3344 (29.6)	8228 (28.4)
	ICU^c^	740 (4.2)	712 (6.3)	1452 (5.0)
**Comorbidities**				
	ECI^d^, median (IQR)	2 (0-5)	3 (1-6)	2 (0-6)
	Hypertension, n (%)	7645 (43.3)	6123 (54.2)	13,768 (47.6)
	Hyperlipidemia, n (%)	5989 (33.9)	3924 (34.7)	9913 (34.2)
	Diabetes mellitus, n (%)	5273 (29.9)	4615 (40.9)	9888 (34.2)
	Coronary artery disease, n (%)	3089 (17.5)	1902 (16.8)	4991 (17.2)
	Congestive heart failure, n (%)	1970 (11.2)	1656 (14.7)	3626 (12.5)
	Stroke or TIA^e^, n (%)	1655 (9.4)	1328 (11.8)	2983 (10.3)
	Dementia, n (%)	1437 (8.6)	845 (7.7)	2282 (8.2)
	Chronic liver disease, n (%)	2424 (13.7)	1878 (16.6)	4302 (14.9)
	CKD-V^f^/ESRD^g^, n (%)	272 (1.5)	565 (5.0)	837 (2.9)
	Respiratory disorders, n (%)	9862 (59.1)	7436 (67.7)	17,298 (62.5)
	Smoking history, n (%)	3115 (17.6)	1833 (16.2)	4948 (17.1)
	Substance use, n (%)	4103 (23.2)	3133 (27.7)	7236 (25.0)
	Alcohol use, n (%)	902 (5.1)	747 (6.6)	1649 (5.7)
	Mental health disorders, n (%)	5331 (32.0)	3227 (29.4)	8558 (30.9)
	Common solid cancer, n (%)	1074 (6.1)	586 (5.2)	1660 (5.7)
	Hematologic malignancies, n (%)	291 (1.7)	147 (1.3)	438 (1.6)
**Geospatial variables, median (IQR)**				
	Florida ADI^h^	5 (3-7)	8 (6-9)	6.0 (4-8)
	Mean PM_2.5_^i^, μg/m^3^	7.35(6.88-7.77)	7.45(7.05-7.96)	7.41 (6.94-7.86)
	Mean NO_2_^j^, ppb^k^	2.73 (1.61-4.63)	3.17(1.88-4.62)	2.87 (1.69-4.63)
	Food environment^l^	4.37 (1.85-4.56)	3.68 (1.85-4.25)	4.10 (1.85-4.25)
	Vacant land^m^	0.22 (0.11-0.48)	0.16 (0.07-0.31)	0.19 (0.10-0.40)
**Unadjusted outcomes, n (%)**				
	Composite outcome	1423 (8.1)	1233 (10.9)	2656 (9.2)
	Death at index encounter	251 (1.4)	238 (2.1)	489 (1.7)

^a^All *P* values were <.001 (except CAD and history of smoking (*P*=.148 and .002, respectively). Independent sample *t* test, Wilcoxon-Mann-Whitney *U* test, or Pearson chi-square test were used wherever appropriate. Values are presented as means (SD), n (%), or median (IQR).

^b^ED: emergency department.

^c^ICU: intensive care unit.

^d^ECI: Elixhauser Comorbidity Score.

^e^TIA: transient ischemic attack.

^f^CKD-V: chronic kidney disease stage 5.

^g^ESRD: end-stage renal disease.

^h^ADI: area deprivation index.

^i^PM_2.5_: particulate matter 2.5.

^j^NO_2_: nitrogen dioxide.

^k^ppb: parts per billion.

^l^Percent students are eligible for reduced-price lunch, 2015.

^m^Percent addresses in the previous quarter with “no-stat” currently in service. “Total No-Stat Addresses” are addresses that can be classified as “No-Stat” for many reasons, including, (1) rural route addresses that are vacant for 90 days or longer and (2) addresses for businesses or homes under construction and not yet occupied addresses.

When examining the outcomes from the XGBoost modeling, the first model, which solely included individual-level variables, had an AUC value of 0.80, indicating reasonably good model performance (Table 3). The balanced accuracy, sensitivity, and specificity were all around the 0.72-0.74 range, suggesting a well-balanced model capable of predicting both positive and negative outcomes with similar accuracy. The outcomes of model 1 subgroups constructed for non-Hispanic White and non-Hispanic Black populations separately exhibited a comparable performance to the overall model 1, suggesting the racial factor has not significantly influenced these particular model outcomes. In model 2, the performance slightly improved when group-level variables were added. The AUC for the general and race-specific models increased to 0.81-0.83. Likewise, the balanced accuracy also increased. There was a marginal reduction in sensitivity for the non-Hispanic White demographic, implying this model predicted fewer true positives for this subgroup. Finally, model 3, which included the ECI (instead of individual comorbidities) and group-level variables from model 2, demonstrated the most optimal performance across all subgroups, with an AUC of 0.82-0.83. Balanced accuracy was also highest for these models, particularly in sensitivity, which indicates these models are better at predicting true-positive outcomes (Table 3).

**Table 3:** Comparative performance metrics of predictive models by race^a^.

Models			AUC^b^		Balanced accuracy		Sensitivity		Specificity
**Model 1, mean (SD)**									
	All	0.80 (0.01)		0.73 (0.01)		0.75 (0.02)		0.71 (0.01)	
	Whites	0.80 (0.01)		0.72 (0.01)		0.74 (0.03)		0.71 (0.01)	
	Blacks	0.80 (0.01)		0.73 (0.01)		0.74 (0.01)		0.72 (0.01)	
**Model 2, mean (SD)**									
	All	0.83 (0.01)		0.74 (0.01)		0.74 (0.02)		0.75 (0.01)	
	Whites	0.82 (0.01)		0.73 (0.01)		0.72 (0.03)		0.75 (0.01)	
	Blacks	0.82 (0.01)		0.74 (0.01)		0.74 (0.01)		0.74 (0.01)	
**Model 3, mean (SD)**									
	All	0.84 (0.01)		0.77 (0.01)		0.81 (0.01)		0.72 (0)	
	Whites	0.84 (0.01)		0.76 (0.01)		0.81 (0.02)		0.72 (0.01)	
	Blacks	0.84 (0.01)		0.76 (0.01)		0.81 (0.01)		0.71 (0.01)	

^a^The table presents comparative performance metrics of predictive models by race, derived from a retrospective cohort study of COVID-19 patients within the OneFlorida Clinical Research Network. The data encompasses adult patients (≥18 years old) diagnosed with COVID-19 between December 1, 2019, and April 28, 2021.

^b^AUC: the area under the receiver operating characteristic curve.

SHAP value analysis in model 1 provided insight into the impact of individual-level variables on predicting COVID-19 outcomes (Figures S2 and S3 in Multimedia Appendix 1 [44-47]). Overall, comorbid conditions such as diabetes mellitus type 2, hypertension, CHF, respiratory disorders, and chronic liver disease consistently ranked the highest across the models and contributed positively to predicting the outcome. Sociodemographic factors such as age and sex ranked consistently high in all models; old age and being female contributed positively to predicting the outcome. BMI and smoking history were important predictors but ranked lower than age and sex. Notably, comorbid conditions such as CHF, rheumatological disorders, and chronic kidney disease stage 5/end-stage renal disease were more significant predictors in the non-Hispanic Black population model, with CHF being the leading predictor.

The incorporation of group-level variables in model 2 yielded additional nuanced findings. Discernible patterns were noted within specific racial subgroups. Comorbid conditions such as diabetes mellitus type 2, hypertension, CHF, and respiratory disorders remained highly ranked and contributed positively to predicting the outcome. Sociodemographic factors such as age and sex remained important predictors, but BMI and smoking history became less important. Group-level variables such as the food environment measure and air pollution measure, PM_2.5_, were among the top predictors. Notably, CHF remained the top predictor for the non-Hispanic Black population model. In addition, Florida ADI ranked relatively higher in the overall and the non-Hispanic White population models compared with the non-Hispanic Black population model. However, the food environment measures ranked higher in the non-Hispanic Black population model than in other models (Figures S4 and S5 in Multimedia Appendix 1 [44-47]).

**Figure 2:**
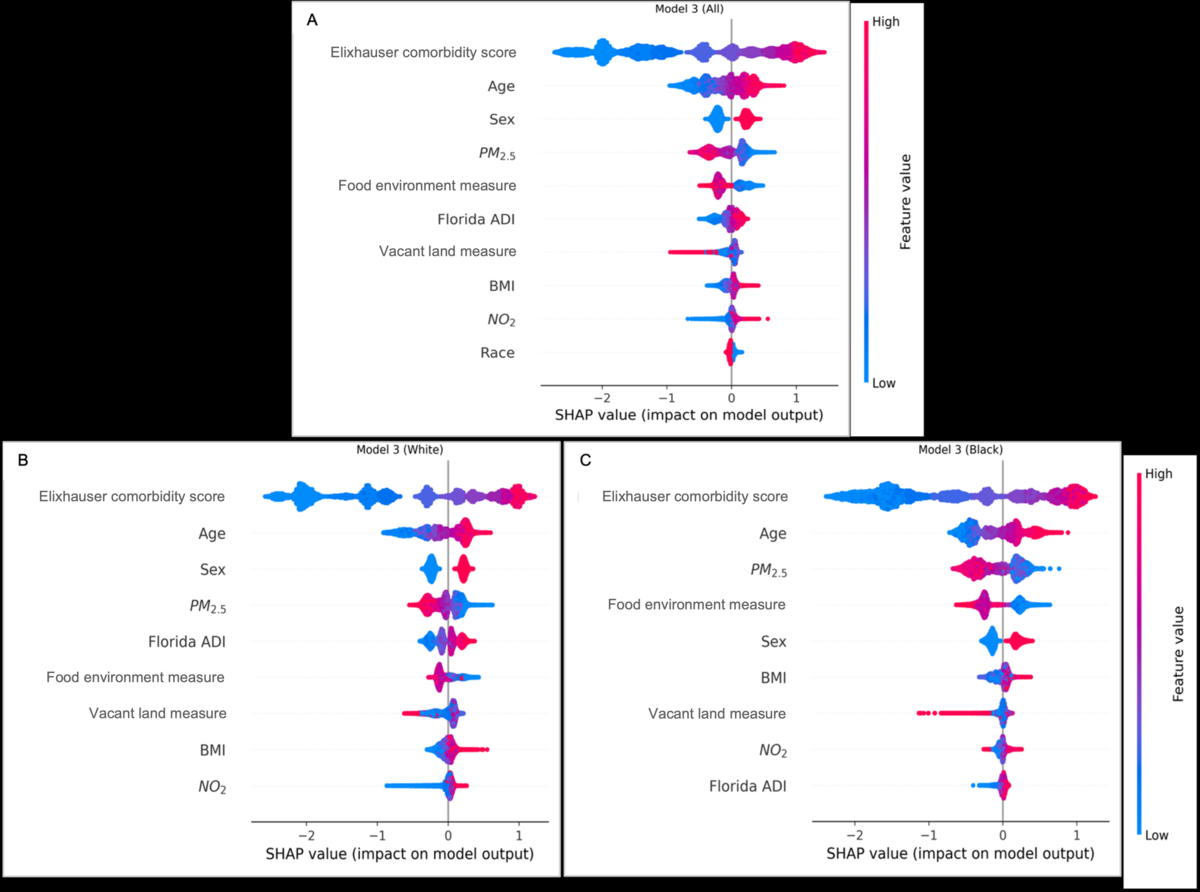
SHAP feature importance model results for outcomes of the COVID-19 pandemic (model 3). ADI: area deprivation index; NO2: nitrogen dioxide; PM2.5: particulate matter 2.5; SHAP: Shapley Additive Explanations.

**Figure 3:**
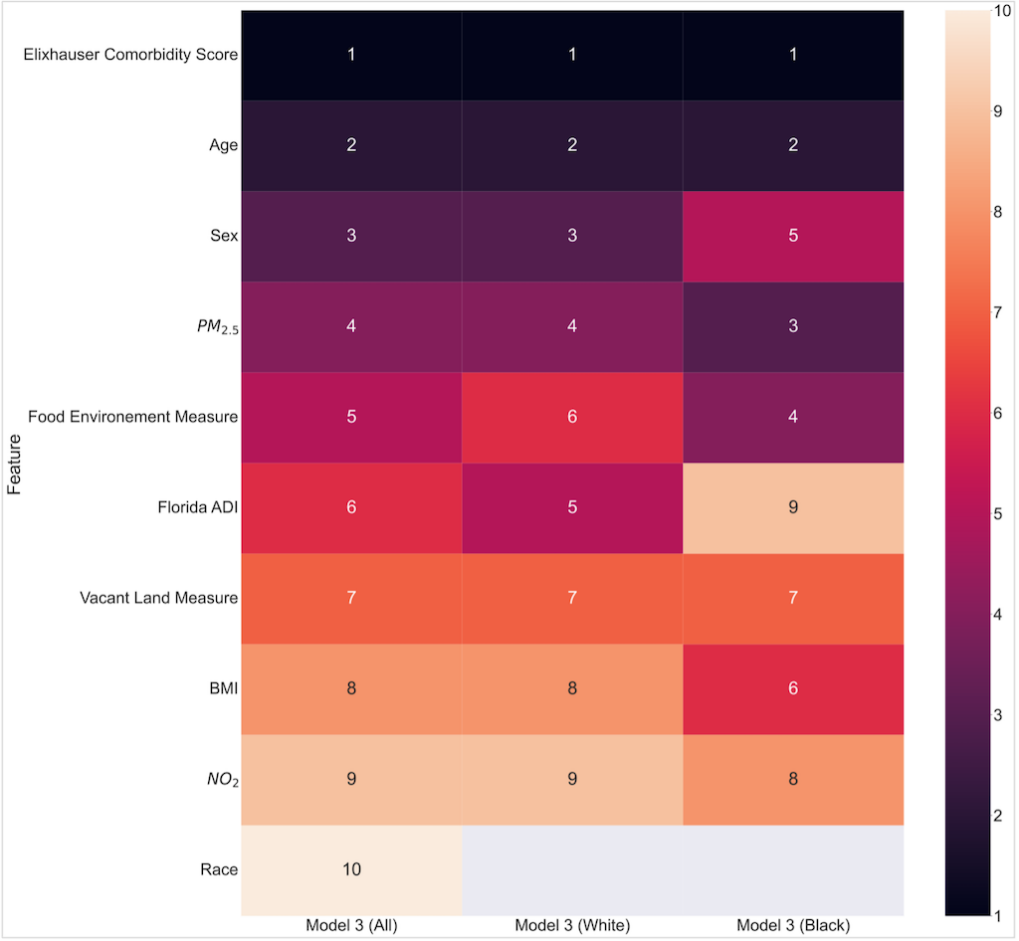
Heat map ranking of Shapley Additive Explanations features importance for outcomes of the COVID-19 pandemic in model 3. ADI: area deprivation index; NO2: nitric oxide; PM2.5: particulate matter 2.5.

Model 3 incorporated comorbidities previously distributed across individual conditions in models 1 and 2, consolidating them into the ECI. This provided a more comprehensive risk measure, where the ECI emerged as the topmost predictor, followed by age, sex, air pollution, and food environment measures. Finally, race generally ranked relatively lower than other predictors in all models (Figures 2 and 3).

The diagrams represent feature importance, arranged from top to bottom. Higher priority features are displayed at the top due to their greater influence on model prediction (having higher predictive power). Color coding indicates the value of the feature: red signifies a higher feature value, while blue represents a lower one. Points to the right of the 0 midline suggest a higher likelihood of the predicted outcome, while those to the left indicate a lower probability of the outcome. Figure 2A displays results for the whole study population. Figure 2B displays results for the non-Hispanic White population. Figure 2C displays results for the non-Hispanic Black population.

The heat map ranks features in model 3 (all population, White population, and Black population) by importance, with number 1 being the most important feature. The food environment measure is the percentage of students eligible for reduced-price lunch, 2015; vacant land measure is the percentage of addresses in the previous quarter with “no-stat” currently in service.

Results from the sensitivity analysis (complete cases only without imputation) showed minor differences compared with the primary analysis. ECI, age, and sex remained the top predictors. In the White population, ADI and vacant land measure ranked higher than PM_2.5_, while in the Black population, no change was noted in feature ranking (Figures S6 and S7 in Multimedia Appendix 1 [44-47]). Similarly, when retraining the model using LightGBM, minimal variations were noted compared with the XGBoost model (Figures S8 and S9 and Table S1 in Multimedia Appendix 1 [44-47]).

## Discussion

### Principal Findings

The COVID-19 pandemic exposed substantial racial disparities. Our study explored the intricate relationship between comorbidities, SDOH, and poor outcomes of COVID-19 in non-Hispanic Black patients. We analyzed real-world data using explainable ML methods to better understand these disparities while acknowledging the need for further causal inference and statistical testing to fully substantiate our findings.

Comorbid conditions and ECI were the most important predictors of outcomes of COVID-19, demonstrating the critical role of baseline comorbidities in predicting poor outcomes of COVID-19, irrespective of race. Mainly, CHF was the most important predictor in non-Hispanic Blacks; the reasons for that are multifaceted and complex [52,53]. Patients with CHF who are diagnosed with COVID-19 infection are often older, predominately Black or Hispanic, have a higher prevalence of diabetes and kidney disease, and use more health care resources. This may serve as a proxy for broader health disparities; Black patients, for example, are generally diagnosed with CHF at later stages and experience worse outcomes despite similar symptoms to their White counterparts [54]. Such disparities can be traced back to limited access to preventive care, higher prevalence of CHF risk factors like hypertension and diabetes, and socioeconomic barriers that hinder effective CHF management among Black communities [54].

Similarly, the food environment measure (county-level percent of students eligible for reduced-price lunch) [39] emerged as one of the top 3 predictors of adverse outcomes among Black individuals. This finding aligns with earlier research [20] and may be partly attributed to historically discriminatory US policies that have resulted in lasting economic and ethnic segregation, manifesting as present-day health disparities [55]. Such inequities are evident in the prevalence of “food deserts,” areas with limited access to healthy food, and the increased risk of repeated hospitalizations, including those due to CHF [56]. Despite adjusting for traditional cardiovascular risk factors, residents of these deprived, racially segregated neighborhoods still faced heightened risk for CVD and CHF [57,58]. Contributory factors include limited recreational facilities [59]; poor walkability [60,61]; and scarce availability of fresh, nutritious foods, particularly in low income and predominantly Black neighborhoods [62].

The feature importance analysis indicated that “race” has the lowest ranking, which may seem counterintuitive at first glance. Nonetheless, this implies that the racial disparities in outcomes of COVID-19 are predominantly linked to inequalities in health conditions, socioeconomic status, and geospatial factors rather than solely to racial identity [2]. This finding should not be construed as minimizing the importance of race; instead, it suggests that the disparities observed across races may be primarily attributable to the unequal distribution of these conditions across racial groups [63]. Race is accepted as a social construct rather than a biological one [64,65]. Health disparities, such as with COVID-19 infection, often arise from enduring inequalities affecting racial and ethnic minorities, notably non-Hispanic Black individuals [63]. Societal and structural dynamics, more than biological distinctions, reinforce these disparities [63]. These discrepancies remain even after considering biological factors and personal health behaviors [2]. The primary drivers are likely the socioeconomic and environmental conditions experienced by diverse racial and ethnic groups. Our model underscores the dominant role of socioeconomic and environmental factors in health outcomes and disparities.

Building on the understanding of race as a key determinant, PM_2.5_ exposure emerged as a notable predictor of health outcomes, ranking fourth for the White population and third for the Black population, ahead of food environment metrics. Previous research links maintained exposure to air pollutants, like PM_2.5_ and NO_2_, with increased COVID-19 mortality [66-68]. Originating mainly from fossil fuel burning, PM_2.5_ could signify heightened pollution exposure in certain demographics, exacerbating outcomes of COVID-19. Environmental findings report greater short- [69] and long-term [70] PM_2.5_ exposure in racial minorities, especially Black individuals, than in White individuals. Following closely is the food environment measure, emphasizing the combined effects of socioeconomic challenges and prolonged PM_2.5_ exposure on health. It highlights the multifaceted roots of health disparities, emphasizing the urgency to tackle both socioeconomic and environmental factors for enhanced public health [71]. Such a holistic perspective is pivotal in understanding spatial variations in outcomes of COVID-19 due to interconnected biological, clinical, socioeconomic, and environmental factors [9,72].

Interestingly, the ADI ranked last in the Black-only model, whereas it came sixth in the White-only model (model 3). This discrepancy does not inherently imply that ADI is inconsequential for the black population but rather that it may interact with other determinants in complex ways. It suggests that although socioeconomic factors are crucial in determining health outcomes, their significance may vary by race. These differences emphasize the importance of considering race-specific factors when analyzing outcomes of COVID-19 and the need for individualized interventions. The higher ranking of the ADI for the White population reflects the well-established relationship between socioeconomic status and health outcomes [34,35]. As measured by the ADI, lower socioeconomic status may contribute to poor health outcomes in this group due to limited access to health care, poorer education, and increased exposure to environmental pollutants and stressors [34,35]. However, the lower ranking of the ADI in the model for the Black population suggests that other factors may be more predictive of outcomes of COVID-19 in this racial group. Another possible explanation is that there is less variation in ADI among Black populations and a greater proportion of Black populations with higher ADIs. The varying ADI values across different socioeconomic levels enable ADI to serve as a strong predictor of outcomes in White populations. In contrast, the higher concentration of individuals with higher ADIs limits the discriminatory power of ADI in this model for Black populations.

Furthermore, in the model focusing on Black individuals, the food environment measure, indicative of food security and nutrition, ranked higher than ADI, a typical socioeconomic marker. This suggests that factors like food insecurity and neighborhood conditions may have a heightened influence on health outcomes in predominantly Black communities [56]. Research also points to racial differences in stress responses, further influencing health outcomes [73]. Importantly, the persisting impact of systemic racism could contribute to poorer health in Black communities [74], irrespective of socioeconomic factors, which may account for the lower ranking of ADI in the Black-specific model.

### Limitations

The study has several limitations. The findings in this study should be considered as part of a broader discussion on racial disparities in health. We acknowledge the need for further causal inference and statistical testing to fully substantiate our observations. The data might not necessarily apply to other states with different demographics or health disparities than Florida. Using EHR data for research is subject to informatic challenges and disadvantages [75]. Race, as used in our study, was self-reported and extracted from EHR. While this method of racial identification is standard in epidemiological research, it comes with inherent challenges that may affect the accuracy and interpretability of the findings. The study could not assess the outcomes of patients who did not require hospitalization or experienced mortality outside the clinical setting. Furthermore, although the XGBoost algorithm has a low risk of overfitting, the lack of an external validation cohort undermines the generalizability of our model.

Similarly, interpreting features’ importance from ML models, such as XGBoost, presents inherent challenges compared with traditional statistical approaches. These outputs do not offer conventional statistical significance measures and can be highly sensitive to model specification and training data characteristics. Also, the models’ evaluation metrics have limitations. The AUC might not fully capture the model’s performance when there is a significant class imbalance or different types of misclassifications vary. Balanced accuracy might not always reflect the practical significance of prediction errors. Sensitivity and specificity can provide a misleading picture of model performance if not considered together, especially in datasets with imbalanced class distributions.

Finally, associations obtained from part of the data at the county level may not reflect individual associations.

In conclusion, our study demonstrates the critical role of model constructs and assumptions in estimating health-related associations, advocating for frameworks that better account for data behaviors. Using a comprehensive ML approach that integrates individual- and group-level exposomic health associations, we used sequential modeling and universal Shapley effect plots for objective comparisons. Our findings emphasize the complexities of health inequalities, particularly persistent racial disparities, and stress the need for multidimensional strategies to address them. Interpretable ML serves as a valuable adjunct to traditional statistical methods, revealing nuanced patterns that can inform resource allocation and policy development for outcomes of COVID-19. Further research is required to clarify the influence of these variables and their contributions to racial health disparities.

## Appendix

Multimedia Appendix 1 Supplementary material.

## Abbreviations

ADI: area deprivation index
AUC: area under the receiver operating characteristic curve
CHF: congestive heart failure
CV: cross-validation
ECI: Elixhauser combined comorbidity index
HCUP: Healthcare Cost and Utilization Project
ML: machine learning
NO2: nitrogen dioxide
PM2.5: particulate matter 2.5
SDOH: Social Determinants of Health
SHAP: Shapley Additive Explanations
XGBoost: Xtreme Gradient Boosting

## Data Availability

The datasets analyzed during this study are not publicly available due to institutional review board protections and privacy considerations. However, the data may be accessed upon reasonable request through the OneFlorida Research Consortium. Information on the data request process is available on the OneFlorida Clinical Research Network’s website.
