# Supplementary material for: Identifying Potential Factors Associated With Racial Disparities in COVID-19 Outcomes: Retrospective Cohort Study Using Machine Learning on Real-World Data
Source: JMIR Public Health Surveill. 2024 Sep 26;10:e54421. doi: 10.2196/54421 (PMC11467607; doi:10.2196/54421)
Supplement: Multimedia Appendix 1 [file publichealth_v10i1e54421_app1.docx]

Table of Contents

[Supplement Methods 1 2](#_Toc162776988)

[Supplement Methods 2 3](#_Toc162776989)

[Supplement Figure 2. SHAP feature importance model results for COVID-19 outcomes (Model 1). 6](#_Toc162776990)

[Supplement Figure 3. Heatmap ranking of SHAP feature importance for COVID-19 outcomes in Model 1. 7](#_Toc162776991)

[Supplement Figure 4. SHAP feature importance model results for COVID-19 outcomes (Model 2). 8](#_Toc162776992)

[Supplement Figure 5. Heatmap ranking of SHAP feature importance for COVID-19 outcomes in Model 2. 9](#_Toc162776993)

[Supplement Figure 6. SHAP feature importance model results for COVID-19 outcomes (Model 4). 10](#_Toc162776994)

[Supplement Figure 7. Heatmap ranking of SHAP feature importance for COVID-19 outcomes in Model 4. 12](#_Toc162776995)

[Supplement Figure 8. SHAP feature importance model results for COVID-19 outcomes (Model 3) using LightGBM. 13](#_Toc162776996)

[Supplement Figure 9. Heatmap ranking of SHAP feature importance for COVID-19 outcomes in Model 3 (main model) using LightGBM. 15](#_Toc162776997)

[Supplement Table 1. Performance of LightGBM on the Model 3 (main model).^a^ 16](#_Toc162776998)

[References 18](#_Toc162776999)

## Supplement Methods 1

We used nested cross-validation (CV) to evaluate our ML models with five outer and inner folds (5x5 nested CV). Nested CV can help ensure rigor and enhance confidence in model generalizability and scalability.^1^ In each outer fold of the nested CV process, one-fifth of the patients’ records served as an independent test set and the rest (four-fifths) as a training set. The outer training set was then equally split into five inner folds in which each inner fold served as an independent validation set, and the other four folds served as an inner training set (inner loop). The inner loop is responsible for model training and hyperparameter tuning (the process of searching for the optimal combination of hyperparameters of the model), while the outer loop is responsible for error estimation and generalization. We used grid search^2^ for hyperparameter tuning, in which exhaustive combinations of the chosen hyperparameters were applied to train the models. All technical training hyperparameter details are displayed below:

| Processor | MacBook with M1 Pro chip |
| --- | --- |
| Software | Python (v3.10.4), sklearn (v1.1.2) |
| Model | eXtreme Gradient Boosting (XGBoost) |
| Max_depth | [3, 4, 5, 6] |
| Learning_rate | [0.01, 0.05, 0.1, 0.3] |
| Gamma | [0, 0.1, 0.2, 0.3] |
| Subsample | [0.5, 0.7, 0.9] |
| Colsample_bytree | [0.5, 0.7, 0.9] |
| Reg_alpha | [0, 0.1, 0.5, 1] |
| Reg_lambda | [0, 0.1, 0.5, 5] |
| Min_child_weight | [1, 3, 5, 7] |
| N_estimator | 500 |

## Supplement Methods 2

SHapley Additive exPlanations (SHAP) values are especially adept at elucidating the functioning of intricate, non-linear ensemble models such as XGBoost.^3^ These values are calculated by assessing how a feature's alteration from a baseline value impacts the model's output, and summing up all such values offers a measure of deviation from a baseline prediction. This framework not only allows for individual feature importance evaluation but also provides a global view by aggregating feature influences across the dataset.^4^ For enhanced interpretation, we employed various visual formats, such as global interpretation summary plots, to depict feature importance and impact. These visual tools also showed the directionality and magnitude of each feature’s contribution to outcomes, thereby shedding light on how these features differentially affect predictions across racial groups. Our analysis included the calculation of SHAP values for all the patients in the test set in each outer fold. After five iterations (five outer folds), each patient was assigned a SHAP value for each feature, thereby obtaining a robust understanding of feature influence.


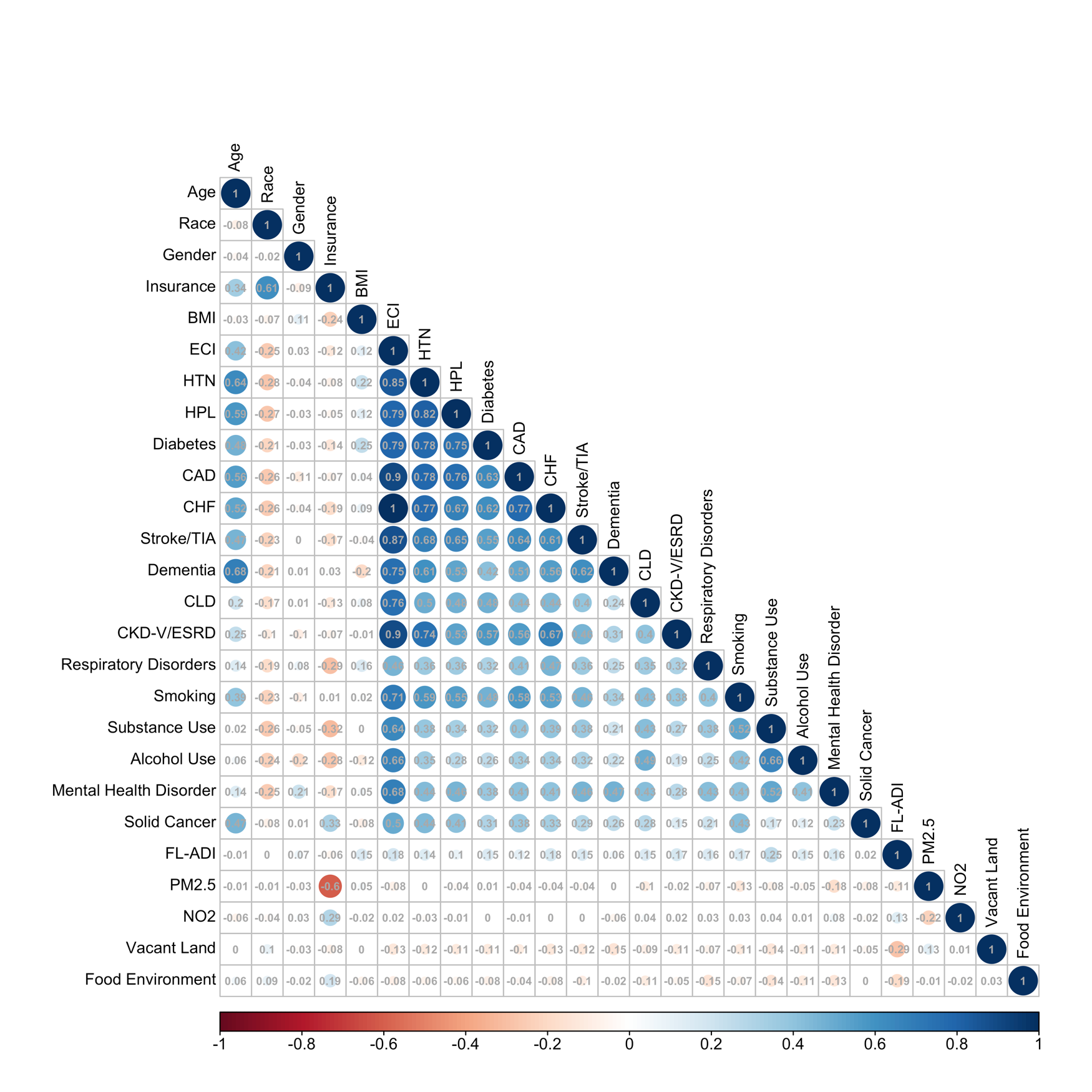
 Supplement Figure 1. Correlations matrix of study variables.^a^

^a^ Correlation coefficients matrix using mixed correlations between variables. Circle size reflects correlation’s strength. The data was derived from the OneFlorida Clinical Research Network and encompasses adult patients (≥18 years old) diagnosed with COVID-19 between December 1, 2019, and April 28, 2021.

Abbreviations: CAD, Coronary Artery Disease; CHF, Congestive Heart Failure; CKD-V, Chronic Kidney Disease stage-5; CLD, Chronic Liver Disease; ESRD, End-Stage Renal Disease; ECI, Elixhauser comorbidity score; FL-ADI, Florida Area Deprivation Index; NO2, nitric oxide; PM2.5, particulate matter 2.5.

## Supplement Figure 2. SHAP feature importance model results for COVID-19 outcomes (Model 1).


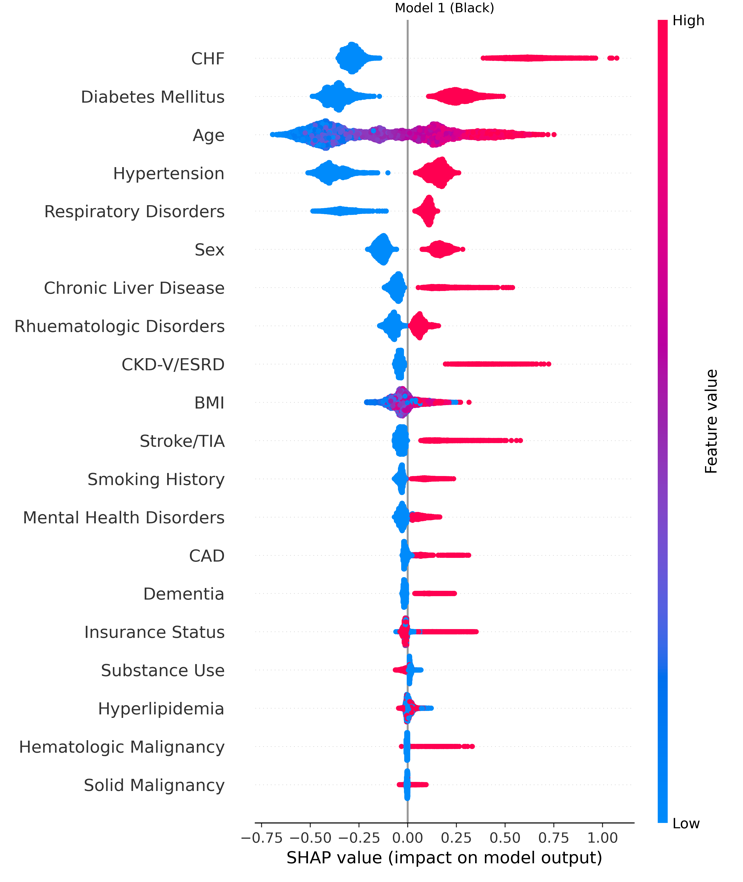

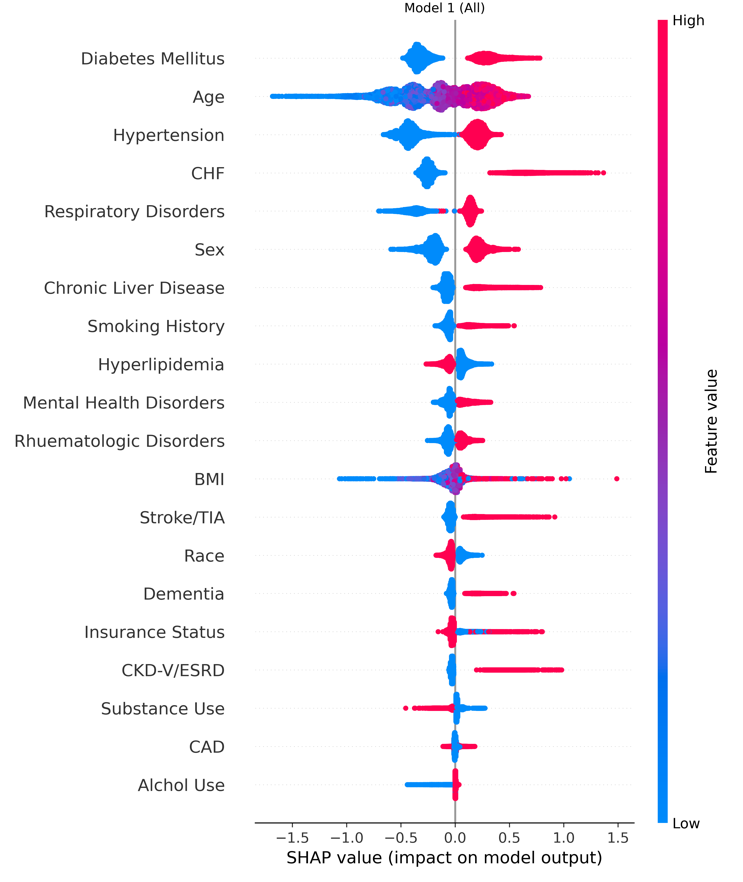

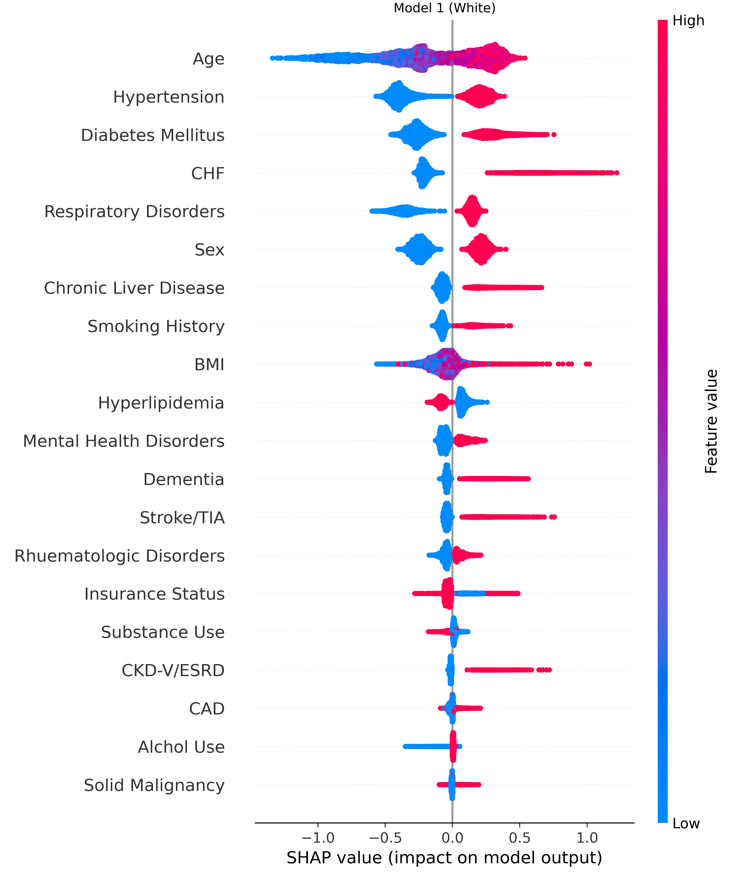

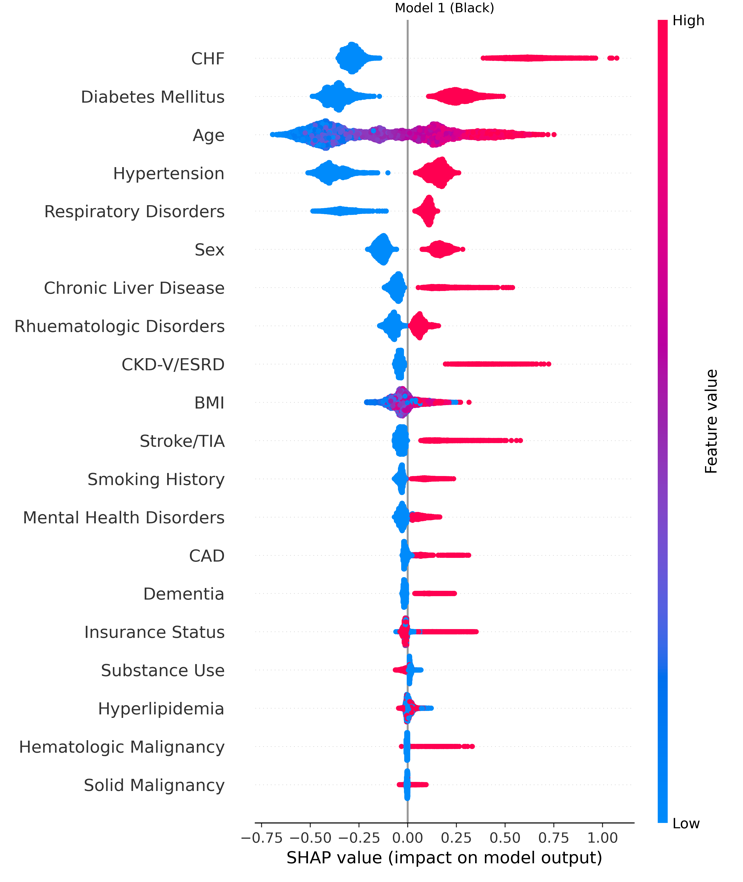


A

B

C

The diagram represents feature importance, arranged from top to bottom. Higher priority features are displayed at the top due to their greater influence on model prediction. Color coding indicates the value of the feature: red signifies a higher feature value, while blue represents a lower one. Points to the right of the 0.00 midline suggest a higher likelihood of the predicted outcome, while those to the left indicate a lower probability of the outcome. A. Displays results for the whole study population. B. Displays results for Non-Hispanic Whites. C. Displays results for Non-Hispanic Blacks. The data was derived from the OneFlorida Clinical Research Network and encompasses adult patients (≥18 years old) diagnosed with COVID-19 between December 1, 2019, and April 28, 2021.

Abbreviations: BMI, body mass index; CAD, coronary artery disease; CHF, congestive heart failure; CKD-V, chronic kidney disease stage-5; ESRD, end-stage renal disease; SHAP, SHapley Additive exPlanations; TIA, transient ischemic attack.


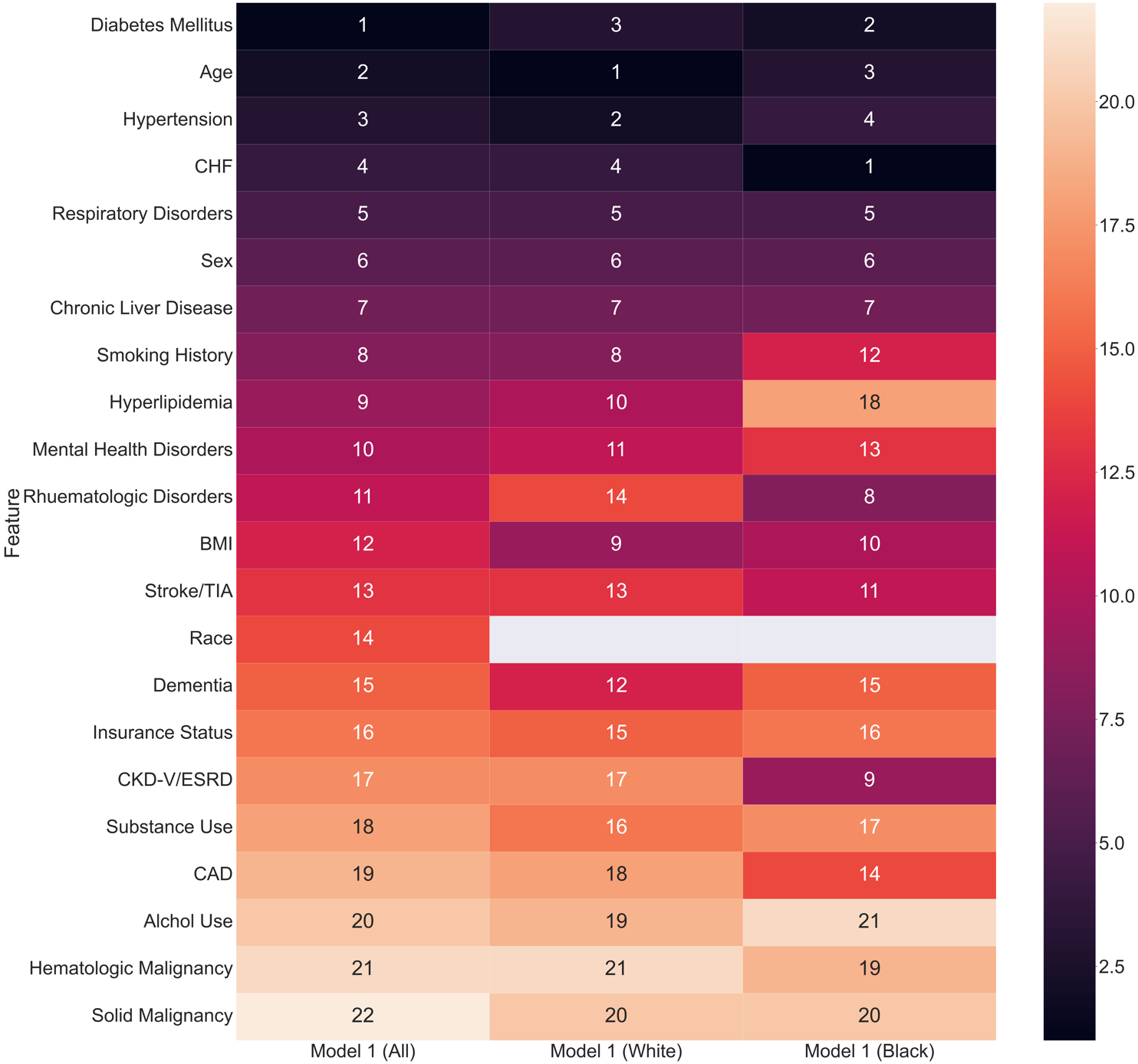
Supplement Figure 3. Heatmap ranking of SHAP feature importance for COVID-19 outcomes in Model 1.

The heatmap ranks features in model 1 (all population, Whites and Blacks) by importance, with number one being the most important feature. The data was derived from the OneFlorida Clinical Research Network and encompasses adult patients (≥18 years old) diagnosed with COVID-19 between December 1, 2019, and April 28, 2021.

Abbreviations: BMI, body mass index; CAD, coronary artery disease; CKD-V, chronic kidney disease stage-5; CHF, congestive heart failure; ESRD, end-stage renal disease; TIA, transient ischemic attack.

Supplement Figure 4. SHAP feature importance model results for COVID-19 outcomes (Model 2).


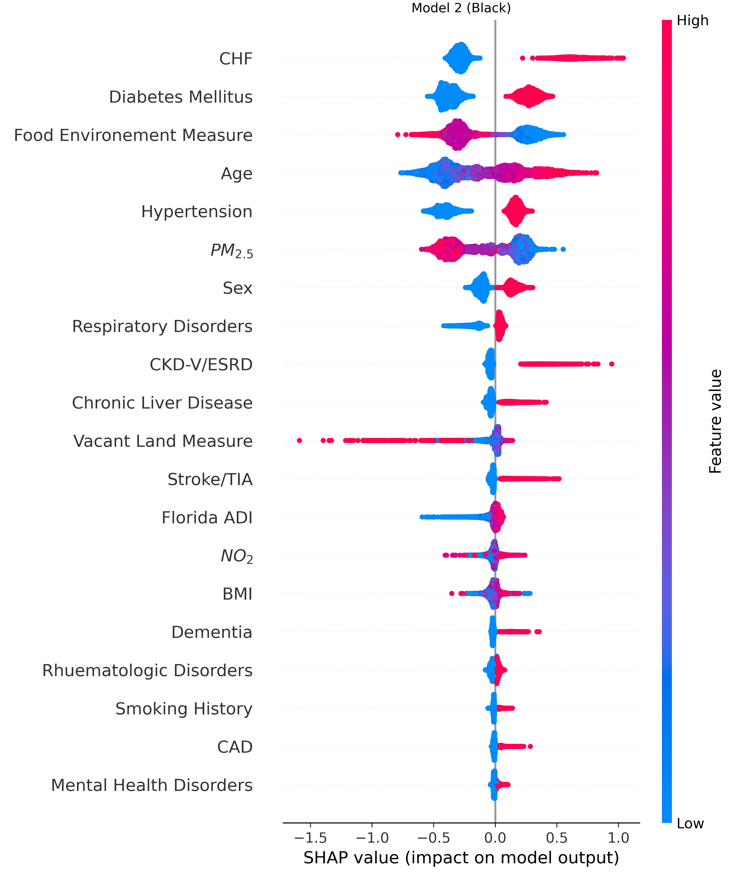

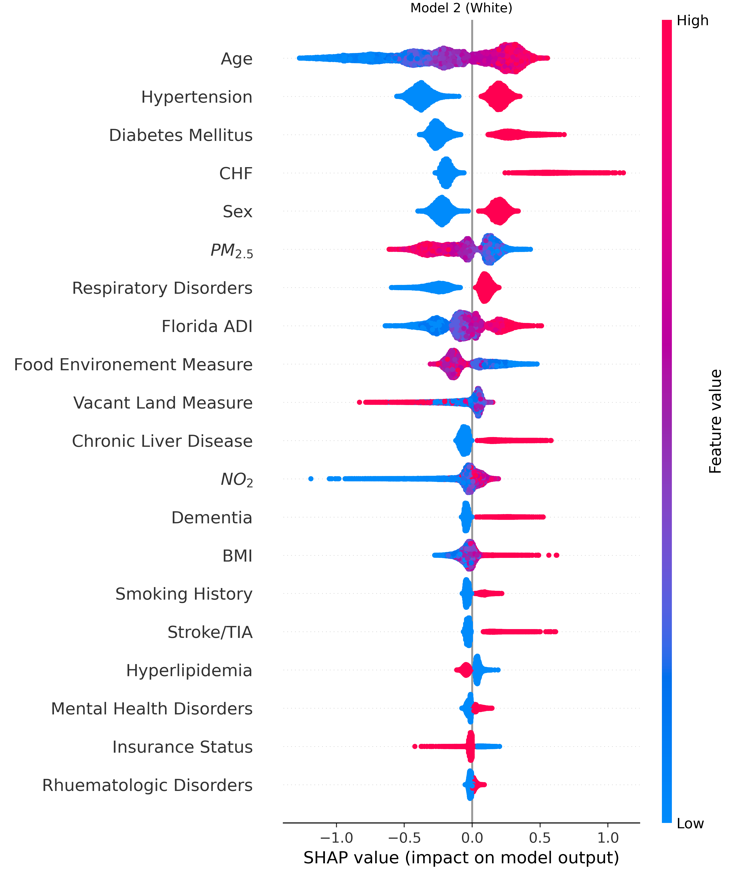

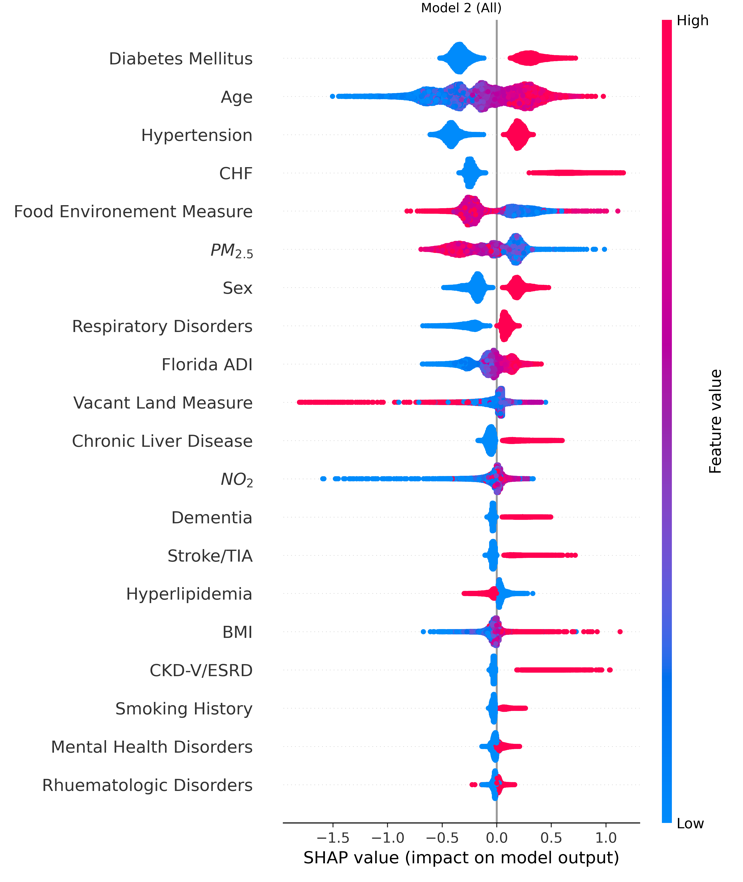

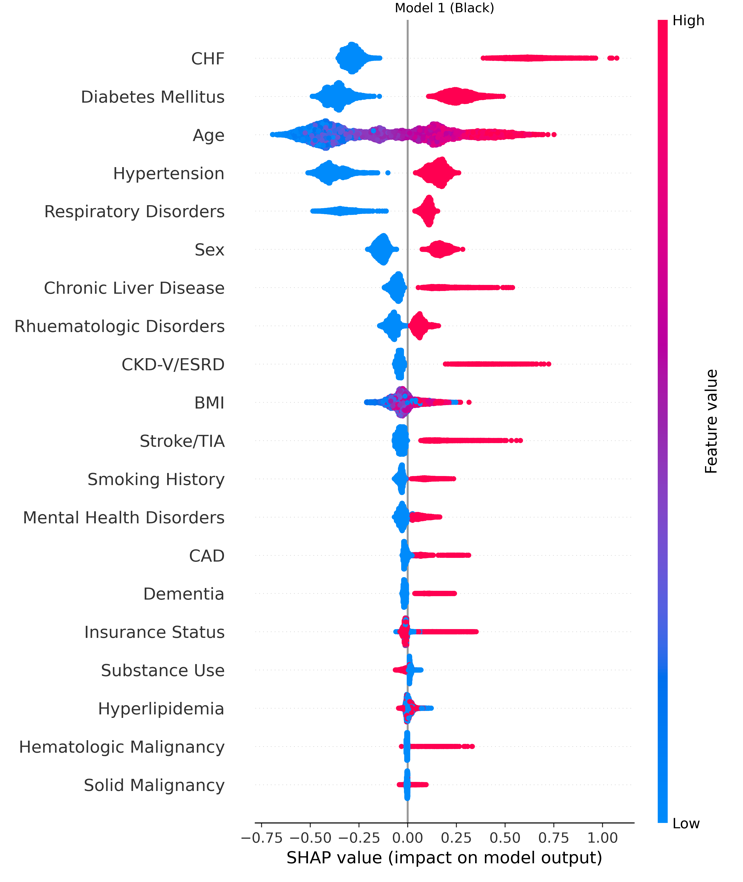


A

B

C

The diagram represents feature importance, arranged from top to bottom. Higher priority features are displayed at the top due to their greater influence on model prediction. Color coding indicates the value of the feature: red signifies a higher feature value, while blue represents a lower one. Points to the right of the 0.00 midline suggest a higher likelihood of the predicted outcome, while those to the left indicate a lower probability of the outcome. A. Displays results for the whole study population. B. Displays results for Non-Hispanic Whites. C. Displays results for Non-Hispanic Blacks. The data was derived from the OneFlorida Clinical Research Network and encompasses adult patients (≥18 years old) diagnosed with COVID-19 between December 1, 2019, and April 28, 2021.

Abbreviations: ADI, area deprivation index; BMI, body mass index; CAD, coronary artery disease; CHF, congestive heart failure; CKD-V, chronic kidney disease stage-5; ESRD, end-stage renal disease; ICU, intensive care unit; NO2, nitrogen dioxide; PM2.5, particulate matter 2.5; SHAP, SHapley Additive exPlanations; TIA, transient ischemic attack.


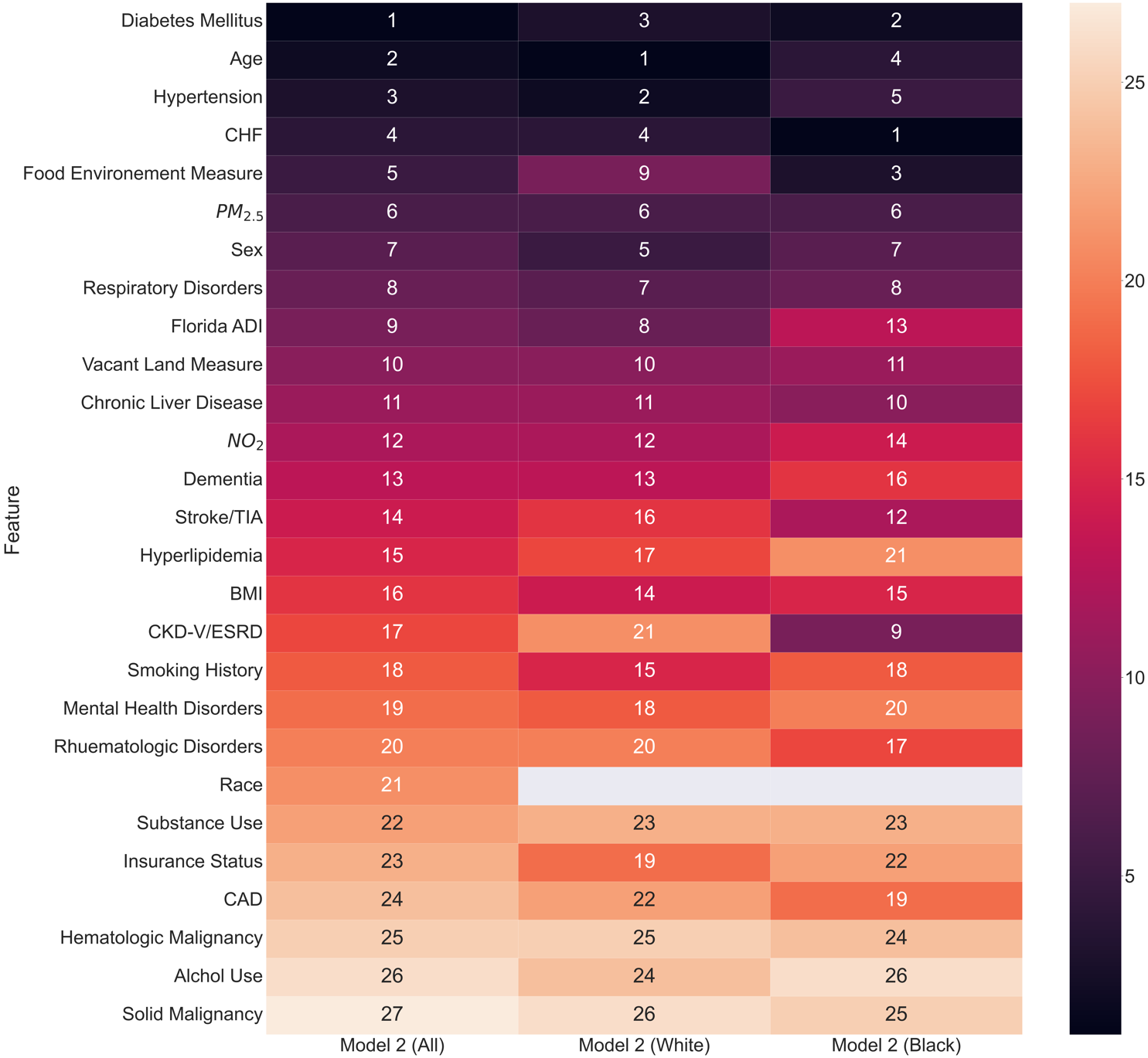
Supplement Figure 5. Heatmap ranking of SHAP feature importance for COVID-19 outcomes in Model 2.

The heatmap ranks features in model 2 (all population, Whites and Blacks) by importance, with number one being the most important feature. The data was derived from the OneFlorida Clinical Research Network and encompasses adult patients (≥18 years old) diagnosed with COVID-19 between December 1, 2019, and April 28, 2021.

Abbreviations: ADI, area deprivation index; BMI, body mass index; CAD, coronary artery disease; CHF, congestive heart failure; CKD-V, chronic kidney disease stage-5; ESRD, end-stage renal disease; ICU, intensive care unit; NO2, nitrogen dioxide; PM2.5, particulate matter 2.5; TIA, transient ischemic attack.

Supplement Figure 6. SHAP feature importance model results for COVID-19 outcomes (Model 4).


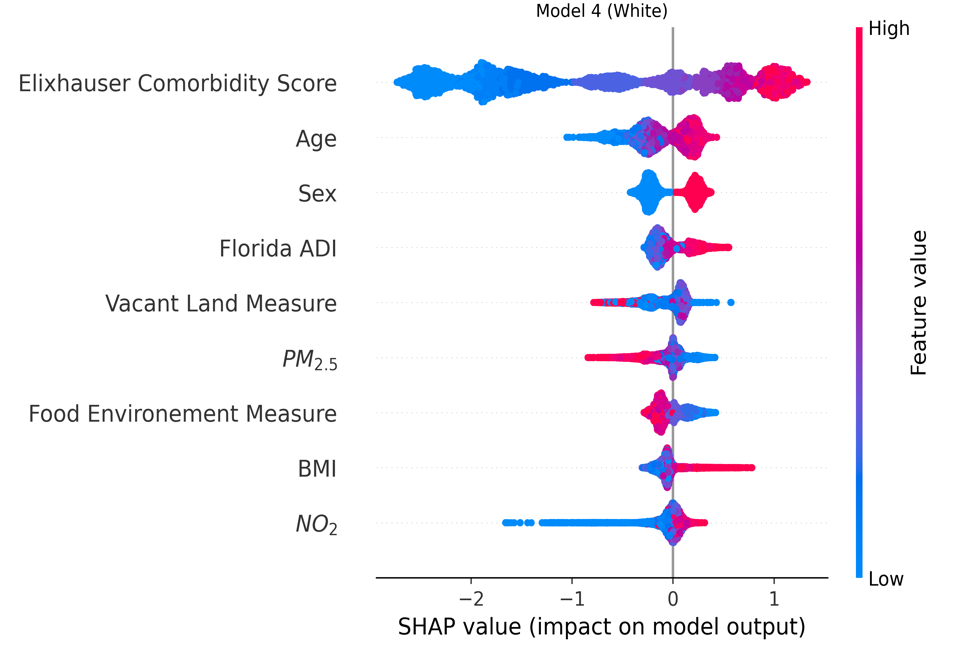

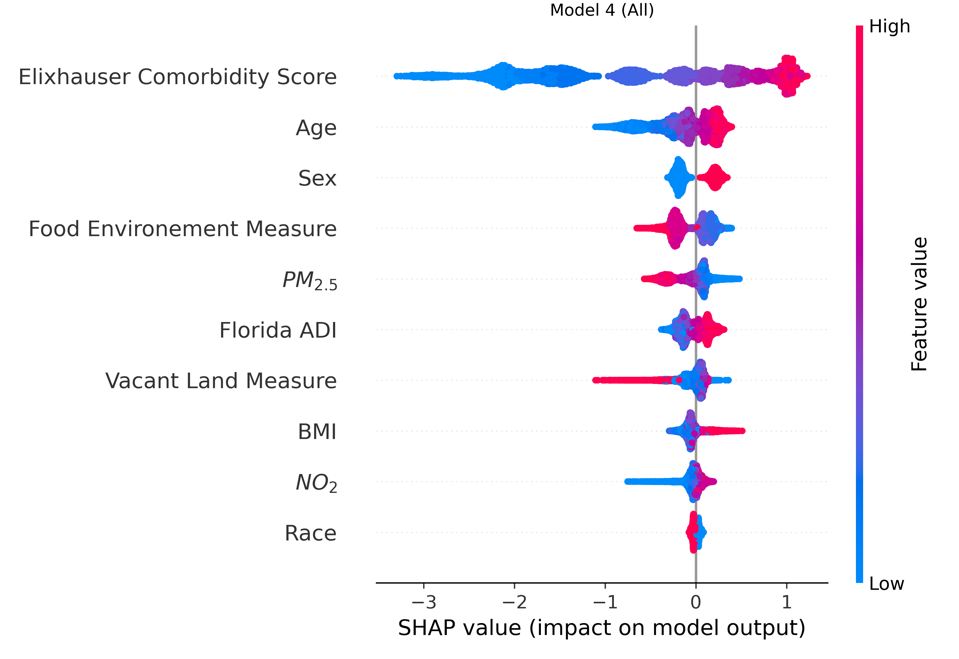

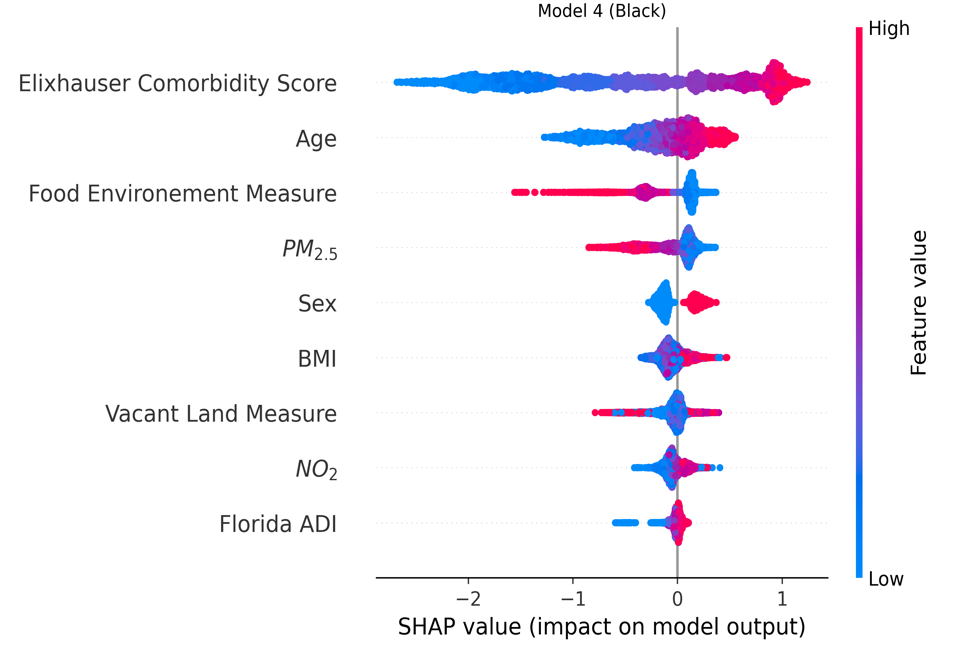

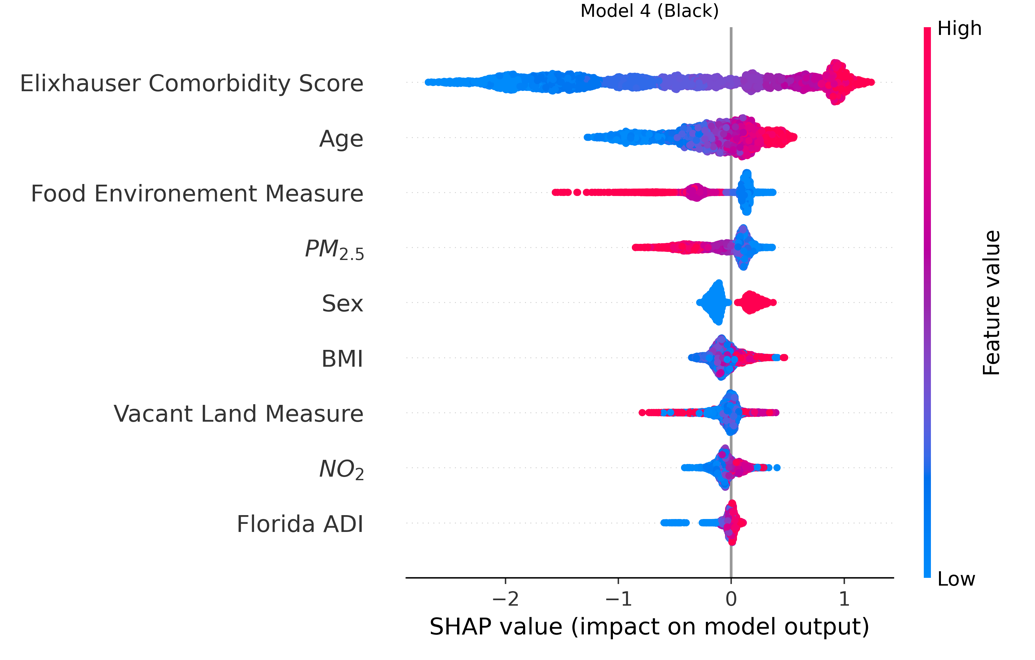

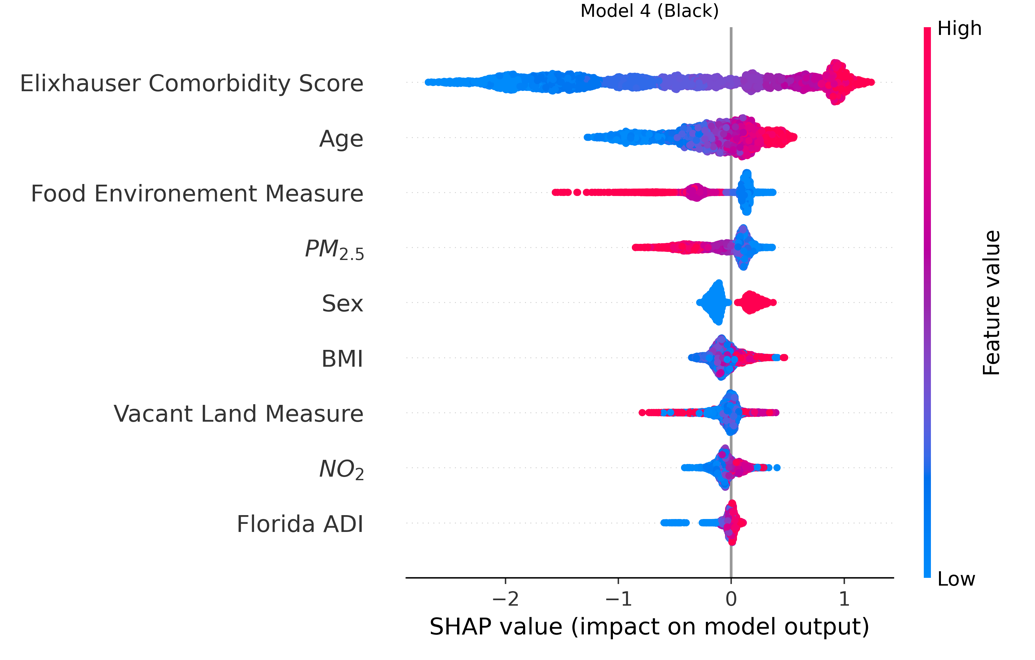


A

B

C

The diagram represents feature importance, arranged from top to bottom. Higher priority features are displayed at the top due to their greater influence on model prediction. Color coding indicates the value of the feature: red signifies a higher feature value, while blue represents a lower one. Points to the right of the 0.00 midline suggest a higher likelihood of the predicted outcome, while those to the left indicate a lower probability of the outcome. A. Displays results for the whole study population. B. Displays results for Non-Hispanic Whites. C. Displays results for Non-Hispanic Blacks. Model 4 is a sensitivity analysis of complete-cases-only data. The data was derived from the OneFlorida Clinical Research Network and encompasses adult patients (≥18 years old) diagnosed with COVID-19 between December 1, 2019, and April 28, 2021.

Abbreviations: ADI, area deprivation index; BMI, body mass index; ECI, Elixhauser comorbidity score; NO2, nitrogen dioxide; PM2.5, particulate matter 2.5; SHAP, SHapley Additive exPlanations.


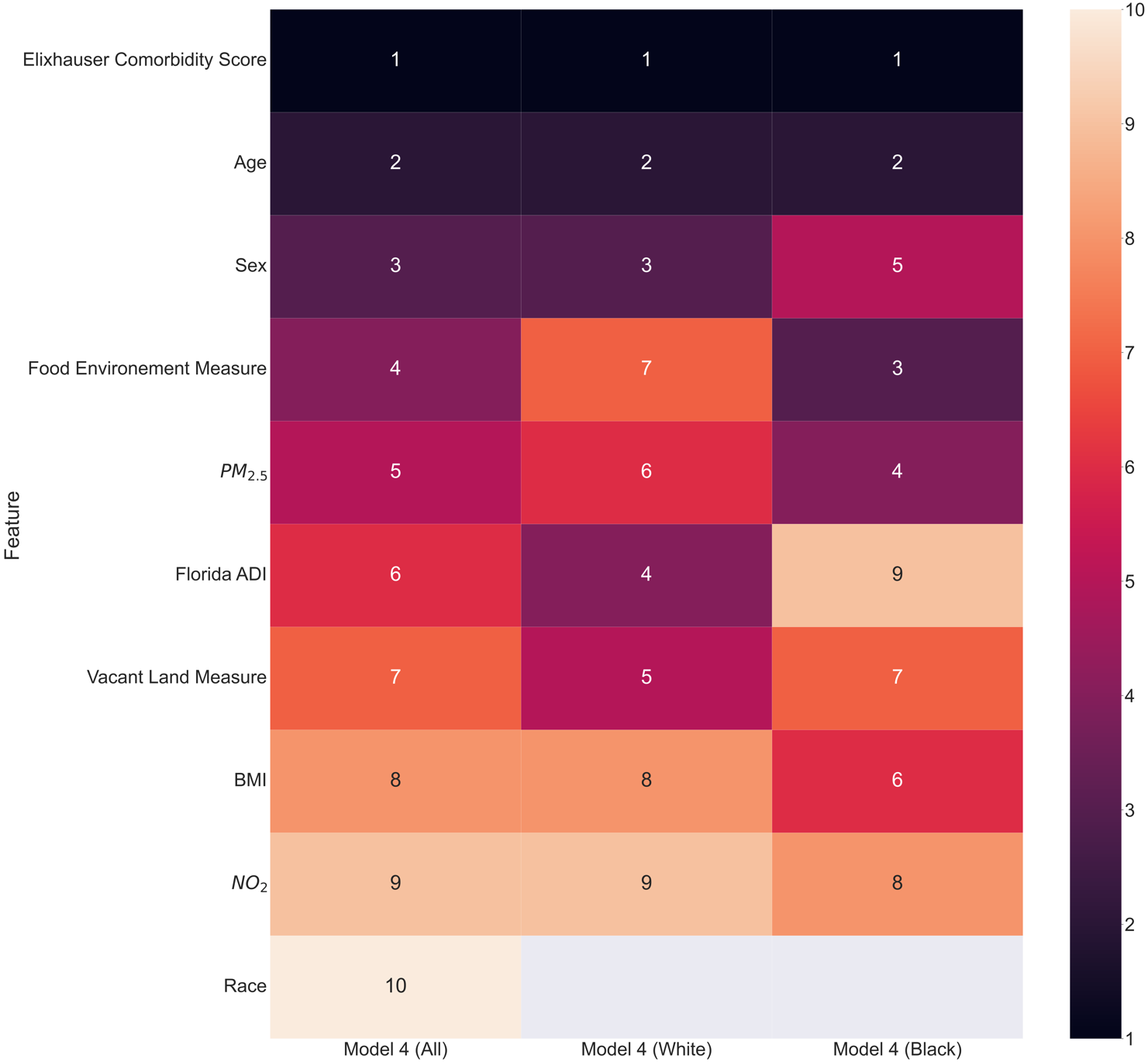
Supplement Figure 7. Heatmap ranking of SHAP feature importance for COVID-19 outcomes in Model 4.

The heatmap ranks features in model 4 (all population, Whites and Blacks) by importance, with number one being the most important feature. Food environment measure = % students eligible for reduced-price lunch, 2015; vacant land measure = % addresses in the previous quarter with ‘no-stat’ currently in service. Model 4 is a sensitivity analysis of complete-cases-only data. The data was derived from the OneFlorida Clinical Research Network and encompasses adult patients (≥18 years old) diagnosed with COVID-19 between December 1, 2019, and April 28, 2021.

Abbreviations: ADI, area deprivation index; ECI, Elixhauser comorbidity score; NO_2_, Nitric Oxide; PM_2.5_, particulate matter 2.5.

Supplement Figure 8. SHAP feature importance model results for COVID-19 outcomes (Model 3) using LightGBM.


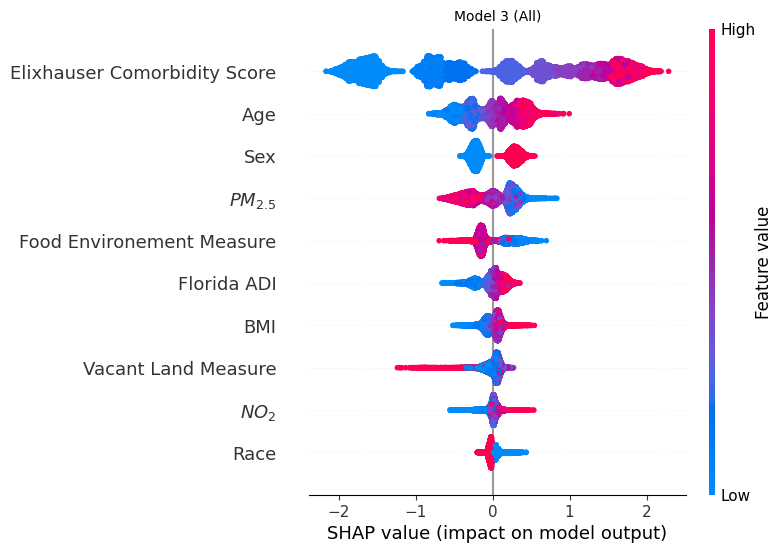

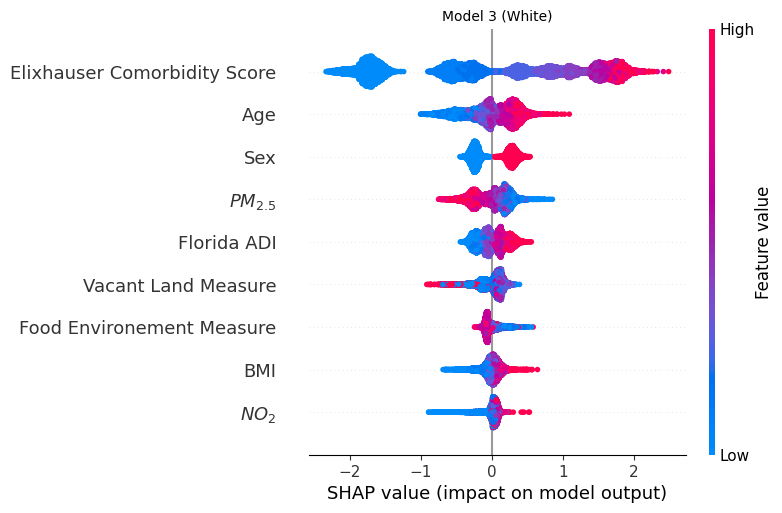

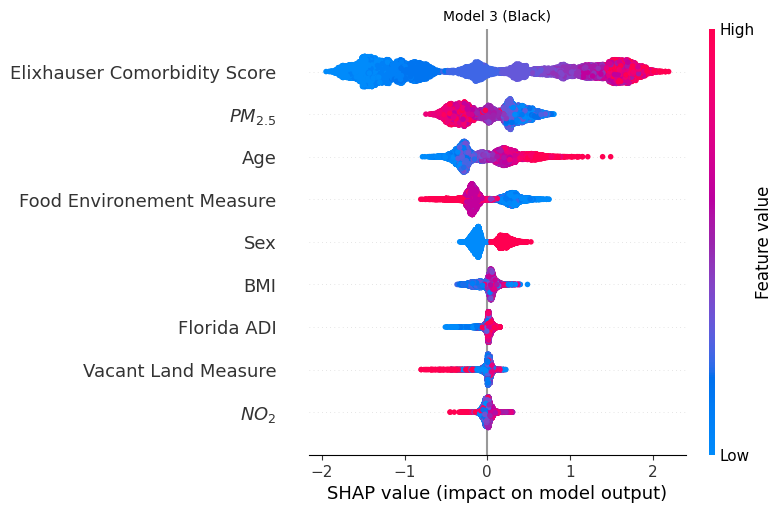


A

B

C

The diagram represents features importance for the LightGBM sensitivity analysis, arranged from top to bottom. Higher priority features are displayed at the top due to their greater influence on model prediction. Color coding indicates the value of the feature: red signifies a higher feature value, while blue represents a lower one. Points to the right of the 0.00 midline suggest a higher likelihood of the predicted outcome, while those to the left indicate a lower probability of the outcome. A. Displays results for the whole study population. B. Displays results for Non-Hispanic Whites. C. Displays results for Non-Hispanic Blacks. Model 4 is a sensitivity analysis of complete-cases-only data. The data was derived from the OneFlorida Clinical Research Network and encompasses adult patients (≥18 years old) diagnosed with COVID-19 between December 1, 2019, and April 28, 2021.

Abbreviations: ADI, area deprivation index; BMI, body mass index; ECI, Elixhauser comorbidity score; LightGBM, Light Gradient Boosting Machine; NO2, nitrogen dioxide; PM2.5, particulate matter 2.5; SHAP, SHapley Additive exPlanations.

Supplement Figure 9. Heatmap ranking of SHAP feature importance for COVID-19 outcomes in Model 3 (main model) using LightGBM.


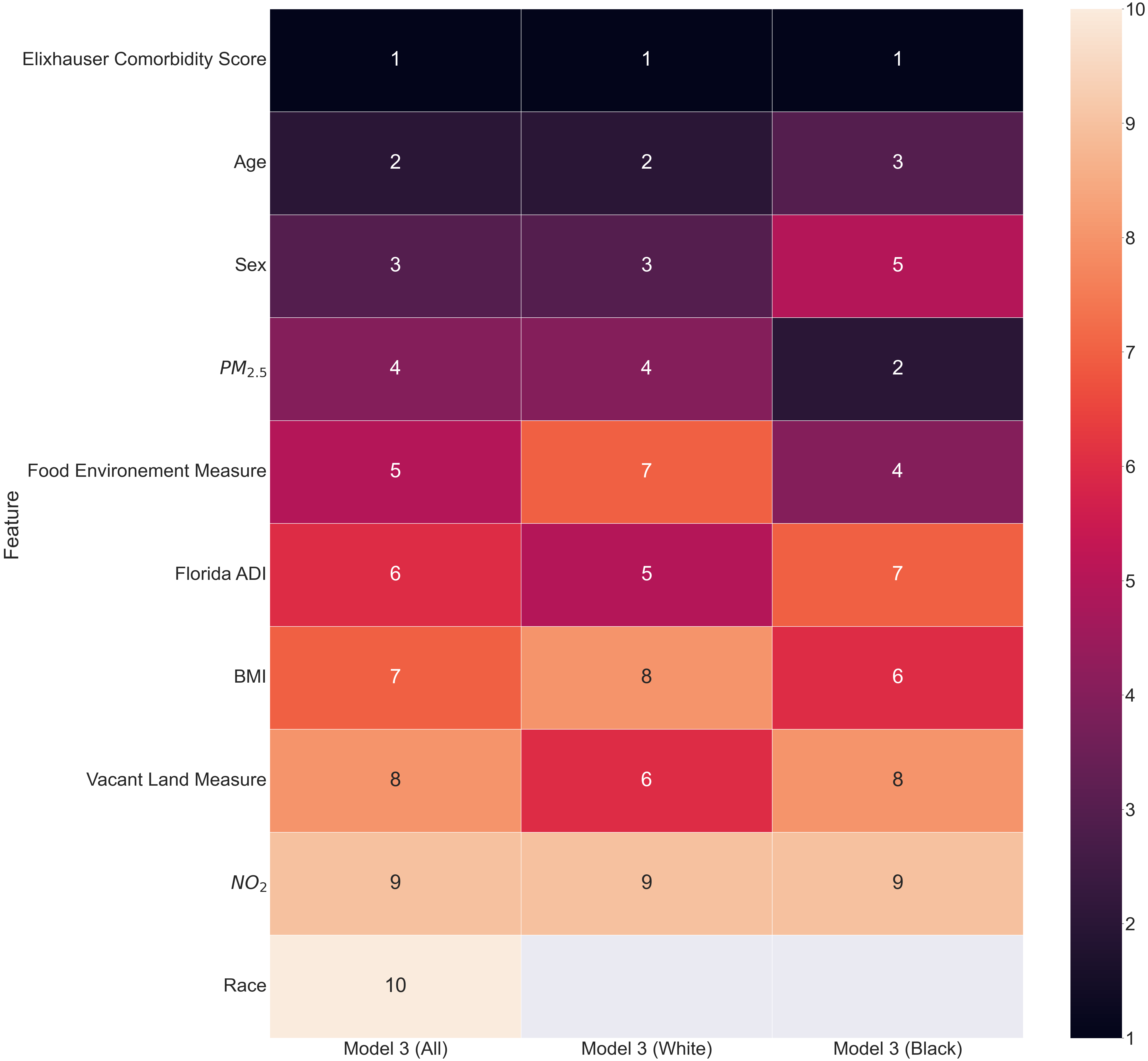


LightGBMT heatmap ranking features in model 3 (all population, Whites and Blacks) by importance, with number one being the most important feature. Food environment measure = % students eligible for reduced-price lunch, 2015; vacant land measure = % addresses in the previous quarter with ‘no-stat’ currently in service. Model 4 is a sensitivity analysis of complete-cases-only data. The data was derived from the OneFlorida Clinical Research Network and encompasses adult patients (≥18 years old) diagnosed with COVID-19 between December 1, 2019, and April 28, 2021.

Abbreviations: ADI, area deprivation index; ECI, Elixhauser comorbidity score; LightGBM, Light Gradient Boosting Machine; NO_2_, Nitric Oxide; PM_2.5_, particulate matter 2.5.

Supplement Table 1. Performance of LightGBM on the Model 3 (main model).^a^

| Model | AUC | Balanced Accuracy | Sensitivity | Specificity |
| --- | --- | --- | --- | --- |
| Model 3-All | 0.84 (0.01) | 0.76 (0.01) | 0.81 (0.01) | 0.72 (0.00) |
| Model 3-Whites | 0.84 (0.01) | 0.76 (0.01) | 0.81 (0.02) | 0.72 (0.01) |
| Model 3-Blacks | 0.83 (0.01) | 0.76 (0.01) | 0.81 (0.02) | 0.71 (0.01) |

^a^ The table presents comparative performance metrics of predictive models by race, derived from a retrospective cohort study of COVID-19 patients within the OneFlorida Clinical Research Network. The data encompasses adult patients (≥18 years old) diagnosed with COVID-19 between December 1, 2019, and April 28, 2021.

Each value represents the performance metric (standard deviation).

Abbreviations: AUC, the area under the receiver operating characteristic curve.

References

1. Cawley GC, Talbot NLC. On over-fitting in model selection and subsequent selection bias in performance evaluation. *Journal of Machine Learning Research*. 2010;11(70):2079-2107.

2. Jiménez ÁB, Lázaro JL, Dorronsoro JR. Finding Optimal Model Parameters by Discrete Grid Search. In: Corchado E, Corchado JM, Abraham A, eds. *Innovations in Hybrid Intelligent Systems*. Advances in Soft Computing. Springer; 2007:120-127. doi:10.1007/978-3-540-74972-1_17

3. Lundberg S. Interpretable Machine Learning with XGBoost. Medium. Published October 6, 2020. Accessed May 25, 2023. https://towardsdatascience.com/interpretable-machine-learning-with-xgboost-9ec80d148d27

4. SHAP Values. Accessed May 25, 2023. https://kaggle.com/code/dansbecker/shap-values
